# Microglia Mediate Synaptic Material Clearance at the Early Stage of Rats With Retinitis Pigmentosa

**DOI:** 10.3389/fimmu.2019.00912

**Published:** 2019-04-26

**Authors:** Juncai He, Congjian Zhao, Jiaman Dai, Chuan Huang Weng, Bai Shi Jiao Bian, Yu Gong, Lingling Ge, Yajie Fang, Hui Liu, Haiwei Xu, Zheng Qin Yin

**Affiliations:** ^1^Southwest Hospital/Southwest Eye Hospital, Third Military Medical University (Amy Medical University), Chongqing, China; ^2^Key Lab of Visual Damage and Regeneration and Restoration of Chongqing, Chongqing, China

**Keywords:** microglia, synapse, rod bipolar cell, retinitis pigmentosa, ectopic neuritogenesis

## Abstract

Resident microglia are the main immune cells in the retina and play a key role in the pathogenesis of retinitis pigmentosa (RP). Many previous studies on the roles of microglia mainly focused on the neurotoxicity or neuroprotection of photoreceptors, while their contributions to synaptic remodeling of neuronal circuits in the retina of early RP remained unclarified. In the present study, we used Royal College of Surgeons (RCS) rats, a classic RP model characterized by progressive microglia activation and synapse loss, to investigate the constitutive effects of microglia on the synaptic lesions and ectopic neuritogenesis. Rod degeneration resulted in synapse disruption and loss in the outer plexiform layer (OPL) at the early stage of RP. Coincidentally, the resident microglia in the OPL increased phagocytosis and mainly engaged in phagocytic engulfment of postsynaptic mGluR6 of rod bipolar cells (RBCs). Complement pathway might be involved in clearance of postsynaptic elements of RBCs by microglia. We pharmacologically deleted microglia using a CSF1 receptor (CSF1R) inhibitor to confirm this finding, and found that it caused the accumulation of postsynaptic mGluR6 levels and increased the number and length of ectopic dendrites in the RBCs. Interestingly, the numbers of presynaptic sites expressing CtBP2 and colocalized puncta in the OPL of RCS rats were not affected by microglia elimination. However, sustained microglial depletion led to progressive functional deterioration in the retinal responses to light in RCS rats. Based on our results, microglia mediated the remodeling of RBCs by phagocytosing postsynaptic materials and inhibiting ectopic neuritogenesis, contributing to delay the deterioration of vision at the early stage of RP.

## Introduction

Retinitis pigmentosa (RP) represents a group of retinal diseases typically attributed to hereditary factors that are caused by mutations predominantly expressed in photoreceptors or retinal pigment epithelial (RPE) cells, that contribute to rod degeneration followed by gradual death of cones (1). Effective treatments that halt or reverse progressive photoreceptor degeneration or RPE loss in patients with RP are currently unavailable. Thus, an investigation of the pathological changes, particularly at the early stage of RP and the development of therapeutic strategies aiming at suppressing its progression are critical. At the early stage of RP, the retina is characterized by the down regulation and mislocalization of proteins of the pre- and postsynaptic machinery in the outer plexiform layer (OPL) in mouse (2–4) and rat (5) RP models. In the rd10 mouse model of RP, which is induced by a mutation in the rod photoreceptor-specific Pde6b gene, photoreceptor stress is accompanied by the loss of axon terminals in photoreceptors and dendritic retraction in bipolar and horizontal cells (6, 7). Dendritic retraction in rod bipolar cells (RBCs) is preceded by a reduction in the immunoreactivity of mGluR6 receptors and their displacement to the cell bodies and axons (8). In Royal College of Surgeons (RCS) rats, a RP model that characterized with defective phagocytosis of photoreceptor outer segments by RPE cells (9), the presynaptic and postsynaptic structures in the OPL are impaired as early as postnatal day 21 (P21) and completely lost at P90 (5). Thus, synaptic dysfunction and loss are the major early pathological changes occurring before the loss of photoreceptors and RPE cells during retinal degeneration (10). However, the mechanisms of synapse loss in RP are not completely understood.

Microglia are the major resident immune cells in the central nervous system (CNS), with well-established immunomodulating properties during the progression of neurodegeneration or injuries (11). Microglia also play an important role in the phagocytic elimination of synaptic elements as part of the widespread pruning of exuberant synaptic connections during CNS development (12, 13). Therefore, microglia have a complex role in shaping maturing or pathological circuits by modifying synapses. During the development of RP, the activation of microglia consists of a targeted migration from the inner retina to the outer nuclear layer (ONL) and a transition from a ramified to an amoeboid morphology (14–17). These activated microglia undergo proliferation (18) and secrete proinflammatory (19) and chemotactic cytokines (20). The release of cytokines by activated microglia induces neurotoxicity and apoptosis signaling, resulting in the degeneration of photoreceptors (21). Inhibition of microglia activation by genetic manipulation (such as Cx3cr1 knockout) (15) or pharmacological suppression (such as minocycline) (19) has shown to delay photoreceptor degeneration. However, the contribution of microglia to synapse loss in the retina of subjects with RP remains unclarified. Circuit remodeling is triggered by early synapse loss and dysfunction in the outer retina (22–24). Therefore, we asked whether the interaction between microglia and synapse regulated synaptic remodeling of neuronal circuits.

In RCS rats, the mutation in the receptor tyrosine kinase Mertk caused the impairment of RPE in phagocytosis photoreceptor outer segments and resulted in the degeneration of photoreceptors (25). As a member of TAM (Tyro3, Axl, and Mertk) family, Mertk and its ligands are also required in the homeostatic, phagocytic clearance of apoptotic cells by microglia (26). So far, the influence of Mertk mutation on the phagocytosis of synapses in the early degenerative retina has not been clarified. In the present study, we explored the roles of microglia in synaptic remodeling of neuronal circuits at the early stage of RP using RCS rat models. Then, a CSF1R inhibitor was used to generically ablate microglia in the RP retina in a sustained manner to validate the effects of microglia on synapse loss and visual function. Using immunohistochemical techniques and electron microscopy, the mechanism underlying the effects of microglia on the synapses of RBCs in the retina were investigated. Our findings revealed a novel role for retinal microglia in engulfment of excess postsynaptic elements and maintenance of RBC structure and function in the RP retina.

## Materials and Methods

### Animals

All experimental procedures were conducted with the approval of the Third Military Medical University Animal Care and Use Committee. RCS-rdy-p+ (RCS rats; P15, 20, 30, 40, and 50; either sex) and RCS-rdy+-p+ rats (Control rats, P15, 20, 30, 40 and 50; either sex) were obtained from the Animal Center of the Third Military Medical University (Chongqing, China). All rats were housed in the animal facility of Southwest Hospital under a 12-h light/dark cycle and fed a standard diet and water.

### Drug Administration

Control or RCS rats were fed a PLX3397-formulated AIN-76A diet (600 p.p.m; 600 mg PLX3397 (Selleckchem, Houston, TX) per kilogram of diet) *ad libitum* to deplete retinal microglia at P15. The control RCS rats were fed a normal AIN-76A diet. Morphological and functional experments were carried out in the rats after continuous depletion of microglia for 5, 15, 25 days respectively.

### Immunohistochemistry

Immunofluorescence staining of frozen tissue sections was performed as previously described (27). Briefly, the enucleated eyecups were fixed with 4% paraformaldehyde (PFA) at 4°C for 15 min and then infiltrated with 30% sucrose overnight at 4 °C. The retinas were embedded in optimal cutting temperature (OCT) compound and cut into 20- or 40-μm-thick sections in the sagittal plane using a freezing microtome. Sections containing the optic nerve were chosen for immunohistochemistry. Sections were permeabilized and blocked with PT1 (PBS containing 0.1% Triton X-100 and 10% donkey or goat serum) at 37°C for 30 min, and then incubated with primary antibodies (Table 1) in PT2 (PBS containing 0.03% Triton X-100 and 5% donkey or goat serum) overnight at 4 °C. After five washes with PBS, sections were incubated with fluorophore-conjugated secondary antibodies in PT2 at 37°C for 1 hour. Nuclei were counterstained with 4′,6-diamidino-2-phenylindole (DAPI; Sigma-Aldrich). Images of immunofluorescence staining were acquired using a confocal microscopy system (Zeiss LSM 780).

**Table 1:** Antibodies used for immunofluorescence staining.

**Antibody**	**Company**	**Titer**	**Species**	**Cat#**
CtBP2	Santa Cruz	1:500	Goat	sc-5966
mGluR6	Neuromics	1:1500	Rabbit	RA13105
IBA1	Wako Chemicals	1:500	Rabbit	019-19741
mGluR6	Abcam	1:500	Guinea pig	ab101864
CD-68	Bio-RDA	1:500	Mouse	MCA341R
Arrestin	Millipore	1:500	Rabbit	AB15282
C1q	Abcam	1:50	Mouse	ab71940
C3	Abcam	1:50	Rabbit	ab11887

TUNEL staining: The terminal deoxynucleotidyl transferase-mediated biotinylated UTP nick end labeling (TUNEL) assay (Roche) was performed according to the manufacturer's specifications to detect the apoptosis of retinal cells.

Analysis of apoptotic cells and surviving cells: For the quantification of cell numbers, all measures were manually performed on images of retina sections using ImageJ software. Total cells (DAPI-positive) and apoptotic cells (TUNEL-positive) in the ONL were counted in three regions (central, right, and left). Sections crossing the optic nerve were selected to strengthen the results. Surviving cells were defined as DAPI-positive nuclei without TUNEL staining: number of surviving cells = number of total cells-number of apoptotic cells. These numbers were averaged for five eyes (from 5 different rats) per group.

Number and morphological score of microglia: Three panoramic sections of the retina per group immunostained for DAPI and IBA1 were captured to quantify the numbers of microglial cells in the different retinal layers. The number of microglia in the total retina or OPL was counted and averaged to obtain the number of cells per 1 mm of the retina for at least three eyes(from 3 different rats) per group. The process morphology was scored as 0 (>15 thick processes with multiple branches), 1 (5–15 thick processes with branches), 2 (1–5 thick processes with few branches), and 3 (no clear processes), using a previously described protocol (12, 28).

### Analysis of Synapses and Ectopic Dendrites in the Retina

Synapses in the OPL were quantified using previously described protocols (29–31). Three to five eyes (from 3-5 different rats) in each group were selected, and 15-20 images with a size of 135 μm × 135 μm were captured from each retinal sample in randomly selected regions. The synapse number was quantified in stacks of 10 optical sections, which were line averaged and collected at 0.28-μm intervals, by projecting a series of five optical sections and counting the number of synapses in each projection volume. Synapses were automatically counted using the ImageJ puncta analyzer program (NIH), and the accuracy of the counts was confirmed manually. Finally, the number of synapses was normalized to the thickness of the inner nuclear layer (INL). The cells in the ONL with positive cytoplasmic staining for cone arrestin and DAPI-stained nuclei were used to quantify the cone cell number. The areas of cone synapse elements were measured using ImageJ software (NIH). The number and length of the ectopic dendrites were quantified using previously described protocols (27). The NeuronJ program in ImageJ software was used to manually trace fluorescently labeled ectopic dendrites from their initial position to their terminus and automatically analyze their number and length.

### Quantification of Microglia Exhibiting Engulfment in the Retina

Engulfment was quantified as previously described (12, 28, 32). For each animal, OPLs in the retinas were chosen for imaging after immunostaining for CtBP2, mGluR6, CD-68 and IBA1. Images were acquired on a confocal microscope at size 135 × 135 μm with 0.2-μm z-steps. The background was subtracted from all fluorescent channels in Z-stacks of optical sections using ImageJ software. Subsequently, 3D volume surface renderings of each z-stack were created using Imaris software (Bitplane). Surface-rendered images were used to determine the volume of the microglia, lysosomes, and synapse elements. The following equation was used to calculate the percent engulfment: volume of lysosomes or synapse elements (μm^3^) /volume of microglial cells (μm^3^). For each eye, 9–12 fields (15–20 microglia) were imaged in the OPLs of retinas, and 3–4 eyes(from 3–4 different rats) in each group were examined.

### Reverse-Transcription Quantitative Polymerase Chain Reaction (RT-qPCR)

The RT-qPCR analysis of the CtBP2 and mGluR6 mRNAs was performed as previously described (27). Briefly, total RNA was extracted from the retinas of rats in each group (Control, Control + PLX, RCS, and RCS + PLX) using TRIzol. Total RNA (approximately 1–2 μg per 20 μl reaction) was reverse transcribed using a PrimeScript®RT Reagent Kit (Takara Bio USA, Mountain View, CA, USA). A SYBR Green qPCR Mix (Dongsheng Biotech, Guangdong, China) was used to perform quantitative PCR on a CFX96 Real-Time PCR System (Bio-Rad, Hercules, CA, USA) according to the manufacturer's instructions. The relative expression of the CtBP2 and mGluR6 mRNAs was normalized to glyceraldehyde 3-phosphate dehydrogenase (GAPDH). All primers were purchased from Sangon Biotech (listed in Table 2).

**Table 2:** Primers used for RT-qPCR.

**Name**	**Sequence**
mGluR6-F	GTGCTAGGTCAACCCTCAAA
mGluR6-R	CTAGAAGAGATCCCAGAGGAGAA
CtBP2-F	AAGGCACGCGGGTACAAAGC
CtBP2-R	CCTGTGATTGCTCGGCGGAT
GAPDH-F	GCCCATCACCATCTTCCAGGAG
GAPDH-R	GAAGGGGCGGAGATGATGAC

### Transmission Electron Microscopy

The retinas from the eyecups were fixed with 0.1 M cacodylate buffer containing 2.5% glutaraldehyde (pH 7.4) for 24 h. After several rinses with PBS, the samples were fixed with 1% osmium tetroxide for 2 h. After several washes with PBS again, the samples were dehydrated with increasing concentrations of acetone and embedded in epoxy resin 618. Uranyl acetate and lead citrate were used to stain the sections. A TECNAI 10 (FEI-Philips, Hillsboro, OR, USA) transmission electron microscope was used to obtain images. Three eyes (from 3 different rats) in each group were selected, and 8–12 images of OPL were captured from each retinal sample in randomly selected regions.

### Electroretinography (ERG)

ERG was performed using previously described methods (27, 33). Briefly, animals were dark-adapted for 12 h and prepared for recording under dim red light (wavelength > 620 nm). Light stimuli were delivered at intensities of −4.5, −2.5, −0.5, −0.02, 0.5 and 1 log(cd·s·m^−2^). Gold wire loops were simultaneously used to record the corneal ERG responses from both eyes. According to the light intensity, the interstimulus interval ranged from 30 to 120 s. The RETIscan system (Roland Consult, Brandenburg, Germany) was used to acquire the data, which were processed using Igor software.

### Statistics

Data were analyzed using an independent two-sample *t*-test, one-way analysis of variance (ANOVA) or two-way ANOVA with SPSS 17.0 statistical software (SPSS Inc., Chicago, IL, USA). The data are presented as means ± the standard deviations (SD). *P*-values less than 0.05 were considered statistically significant.

## Results

### Synapse Disruption and Loss at the Early Stage of Retinal Degeneration in the RCS Rat

**Figure 1:**
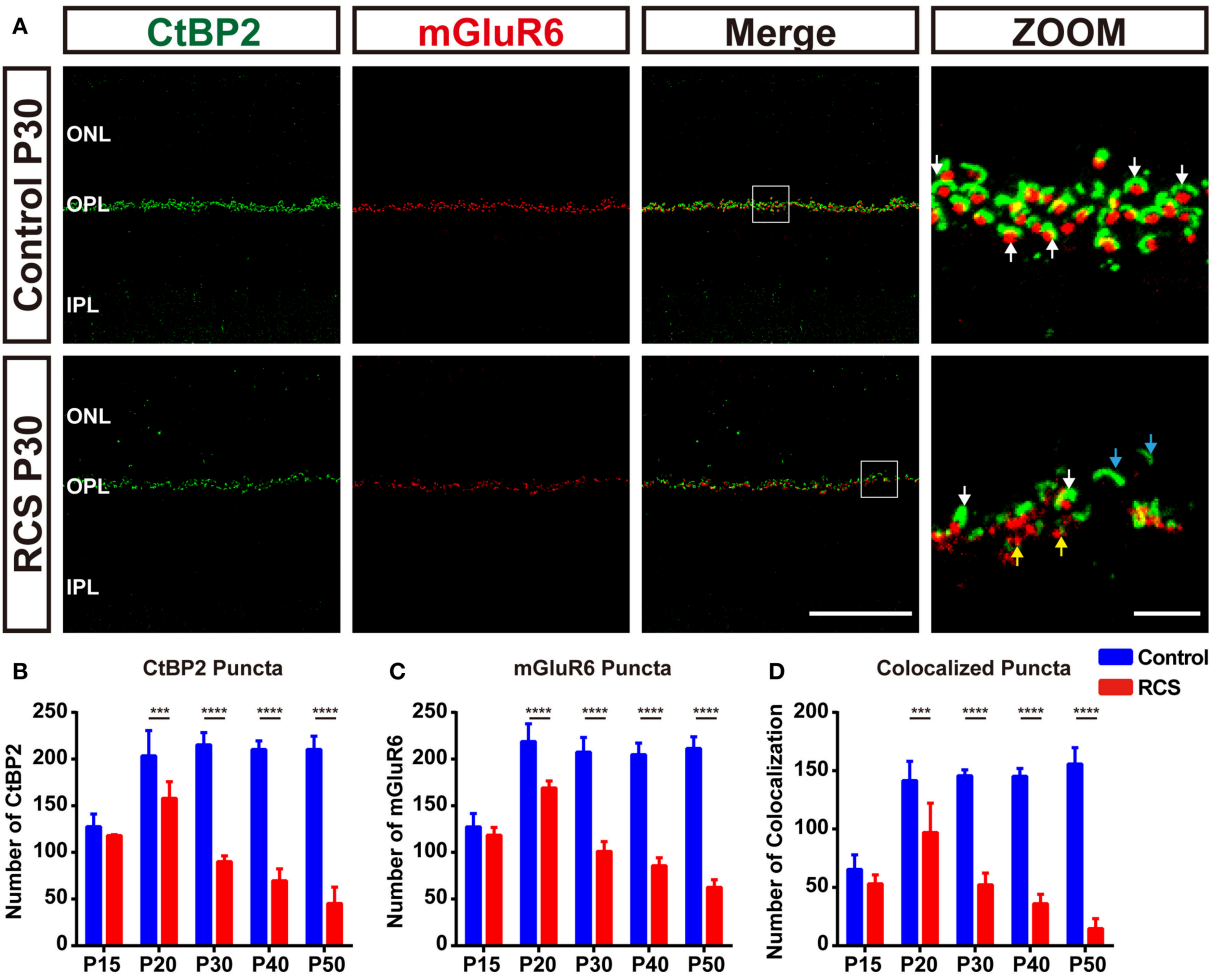
Early synapse loss in the OPL during retinal degeneration in RCS rats. **(A)** Confocal images of CtBP2- (green) and mGluR6-immunoreactive (red) puncta in the retinas of control (upper panel) and RCS rats (lower panel) at P30 showed that the normal structure of synapses in the OPL was lost. White arrows indicate normal rod synapses, cyan arrows indicate unpaired CtBP2-positive puncta, and yellow arrows indicate unpaired mGluR6-positive puncta. **(B–D)** Quantification of synaptic puncta or their apposition in the OPL of control and RCS rats at P15, P20, P30, P40, and P50, indicating the loss of CtBP2, mGluR6 and colocalized puncta per field of view (135 × 135 μm) during retina degeneration. (*N* = 3–5 eyes from different rats, *n* = 15–20 images from each eye). ONL, outer nuclear layer; OPL, outer plexiform layer; IPL, inner plexiform layer; Scale bar, 50 μm **(A)** or 5 μm **(A)**. Bars represent means; error bars represent SD. ^***^p < 0.001, ^****^p < 0.0001 using two-way ANOVA **(B–D)**.

**Figure 2:**
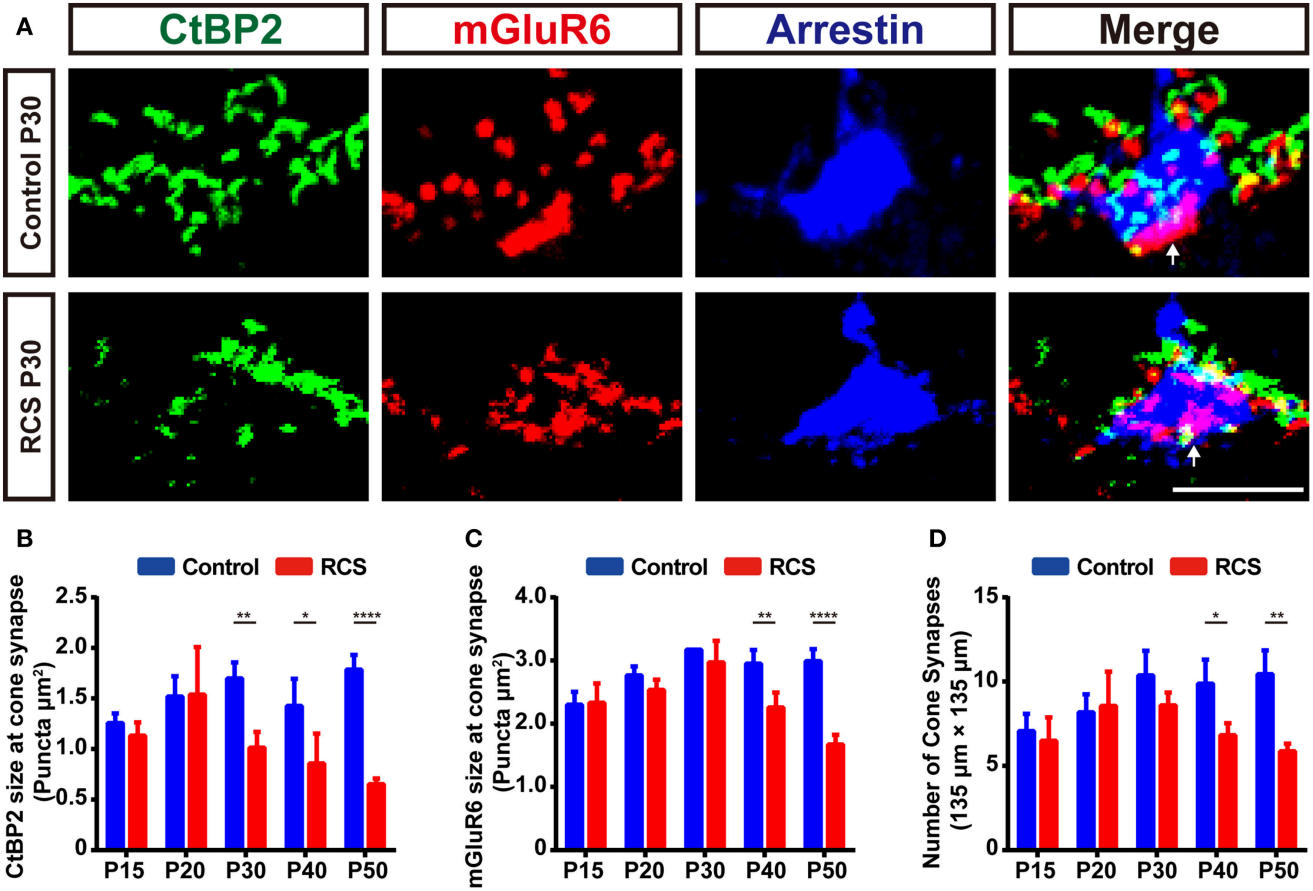
Early cone synapse loss in the OPL during retinal degeneration in RCS rats. **(A)** Immunostaining for CtBP2 (green), mGluR6 (red) and cone arrestin (blue) in the retinas of control and RCS rats at P30. White arrows indicate cone synapses. **(B–C)** Quantification of the changes in the size of cone synaptic puncta positive for CtBP2 and mGluR6 in control and RCS rats at P15, P20, P30, P40, and P50 (*N* = 3–4 eyes from different rats, *n* = 9–15 images from each eye). **(D)** Quantification of the changes in the number of cone synapse in control and RCS rats at P15, P20, P30, P40, and P50 (*N* = 3–4 eyes from different rats, *n* = 9–15 images from each eye). Scale bar, 5 μm **(A)**. Bars represent means; error bars represent SD. ^*^p < 0.05, ^**^p < 0.01, ^****^p < 0.0001 using two-way ANOVA **(B–D)**.

Significant synaptic changes occur at a very early stage in the RCS rat, even prior to any significant loss of photoreceptors, in parallel with the deterioration of ERG responsiveness specifically involving the rod system (5, 34). To determine temporal and spatial characteristics of the synapse changes, we used high resolution confocal microscopy to quantify synapse density in the OPL of RCS rat retinas (Supplementary Figure 1A). CtBP2, a marker of presynaptic ribbons, and mGluR6 were chosen to label the synapses in the OPL. In control rats, rod synapse and cone synapse could be distinguished based on their morphology of presynaptic and postsynaptic pairings. The synapses between rods and RBCs consisted of horseshoe-like, CtBP2-positive presynaptic ribbons, accompanied by a single, dot-like, mGluR6-positive postsynaptic structure; these pairs were distributed in the outermost part of the OPL (Figure 1A and Supplementary Figure 1B). The synapse between a cone and a cone bipolar cell consisted of 3-4 CtBP2-labeled presynaptic structures and a mGluR6-labeled disk-like postsynaptic structure; these pairs were mainly distributed within the innermost part of the OPL (Figure 2A and Supplementary Figures 1F,G).

During retina degeneration, apparent changes in synapse morphology were detected at P30 in RCS rats (Figure 1A and Supplementary Figure 1C), when scattered apoptotic rods were observed in the ONL (35). In the rod synapse, the morphology of the CtBP2-positive ribbons changed from a typical horseshoe to a short rod shape in the retina of RCS rats, while little alterations in mGluR6-positive postsynaptic elements were observed, even when the synapse was lost. The number of CtBP2-positive (Figure 1B), mGluR6-positive (Figure 1C) and colocalized puncta (Figure 1D) decreased modestly but significantly in the retinas of RCS rats at P20 compared with controls. The decrease peaked at P30 and then slowed at P40. However, the number of cone synapses decreased after P40 in RCS rats (Figure 2D). In the cone synapses, the area of presynaptic CtBP2 staining was gradually reduced at P30 (Figure 2B), a time point that was earlier than the reduction in the size of postsynaptic mGluR6 at P40 (Figure 2C). No significant changes in the CtBP2 mRNA level were observed in the retinas of RCS rats (Supplementary Figure 1D), while the level of the mGluR6 mRNA was markedly increased in the retinas of P40 RCS rats compared with control rats (*p* < 0.01) (Supplementary Figure 1E). Our data indicated that these morphological and quantitative changes of synapse in the OPL were mainly limited in the rod–RBC contacts before P40 in RCS rats.

**Figure 3:**
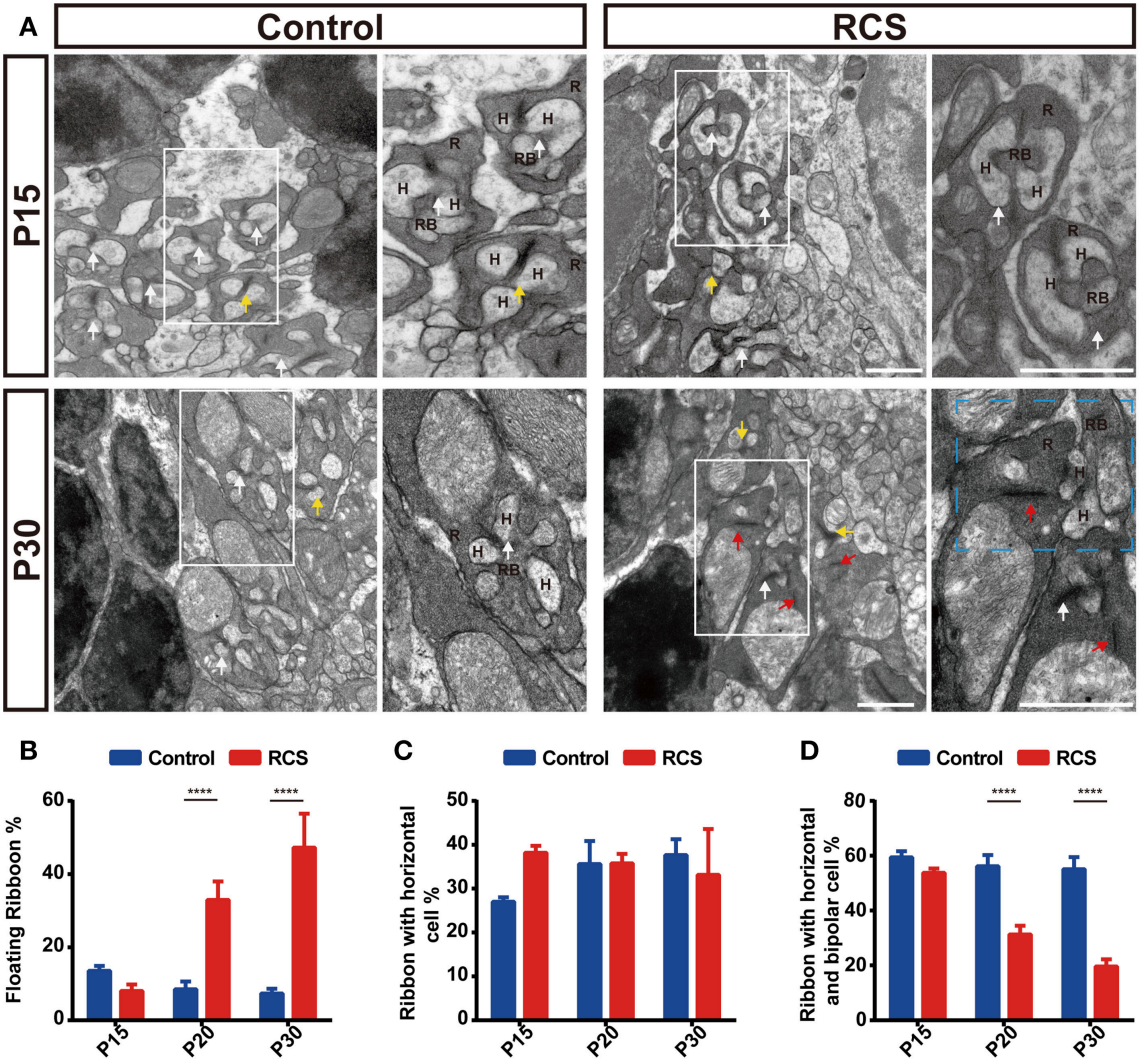
The ultrastructure of rods in ON-RBC synapses in the OPL is disrupted during retinal degeneration in RCS rats. **(A)** Synaptic ultrastructure in the OPL of control and RCS retinas as revealed by transmission electron microscopy at P15 and P30. The cyan rectangle indicates a perturbation of postsynaptic cell dendrite invagination into the photoreceptor terminus. White arrows indicate normal rod synapses, called triads, with three postsynaptic elements (the ribbon was a presynaptic specialization; the three postsynaptic elements invaginated into the base of the photoreceptor such that two horizontal cell and one bipolar cell dendrite are located centrally), red arrows indicate floating ribbons, yellow arrows indicate dyads with only one or two horizontal cell processes opposed to the ribbon. **(B)** Proportion of floating ribbons in control and RCS rats at P15, P20, and P30, indicating that disruption of rod synapse correlated with rod cell stress (*N* = 3 eyes from different rats, *n* = 8–12 images from each eye). **(C)** A difference in the proportion of dyads in RCS rats compared to controls was not observed at P15, P20, and P30 (*N* = 3 eyes from different rats, *n* = 8–12 images from each eye). **(D)** The proportion of normal synapses was significantly decreased during retinal degeneration (*N* = 3 eyes from different rats, *n* = 8–12 images from each eye). R, rod cell; RBC, rod bipolar cell; H, horizontal cell. Scale bar, 1 μm **(A)**. Bars represent means; error bars represent SD. ^****^p < 0.0001 using two-way ANOVA **(B–D)**.

Electron microscopy (ECM) analyses further confirmed that aberrant synapses formed in the retinas of RCS rats, containing only floating ribbons. These floating ribbons in the rod terminal lost their normal localization and were separated from postsynaptic elements (Figure 3A). Significant differences in the proportion of each type of ribbon were not observed in the OPL between controls and RCS rats at P15 (Figures 3A–D), the stage when rod degeneration had not yet started. In P20 and P30 RCS rats, the proportion of floating ribbons to total ribbons increased markedly as retinal degeneration progressed, while the proportion of normal synapse ribbons decreased significantly in RCS rats (Figures 3B–D), suggesting that the decrease in the number of paired CtBP2- and mGluR6-positive structures resulted from rod synapse disintegration. Based on these results, photoreceptor degeneration resulted in synapse loss and disintegration.

### Microglia Increased Phagocytosis and Engulfed Postsynaptic Elements During the Synaptic Perturbation

**Figure 4:**
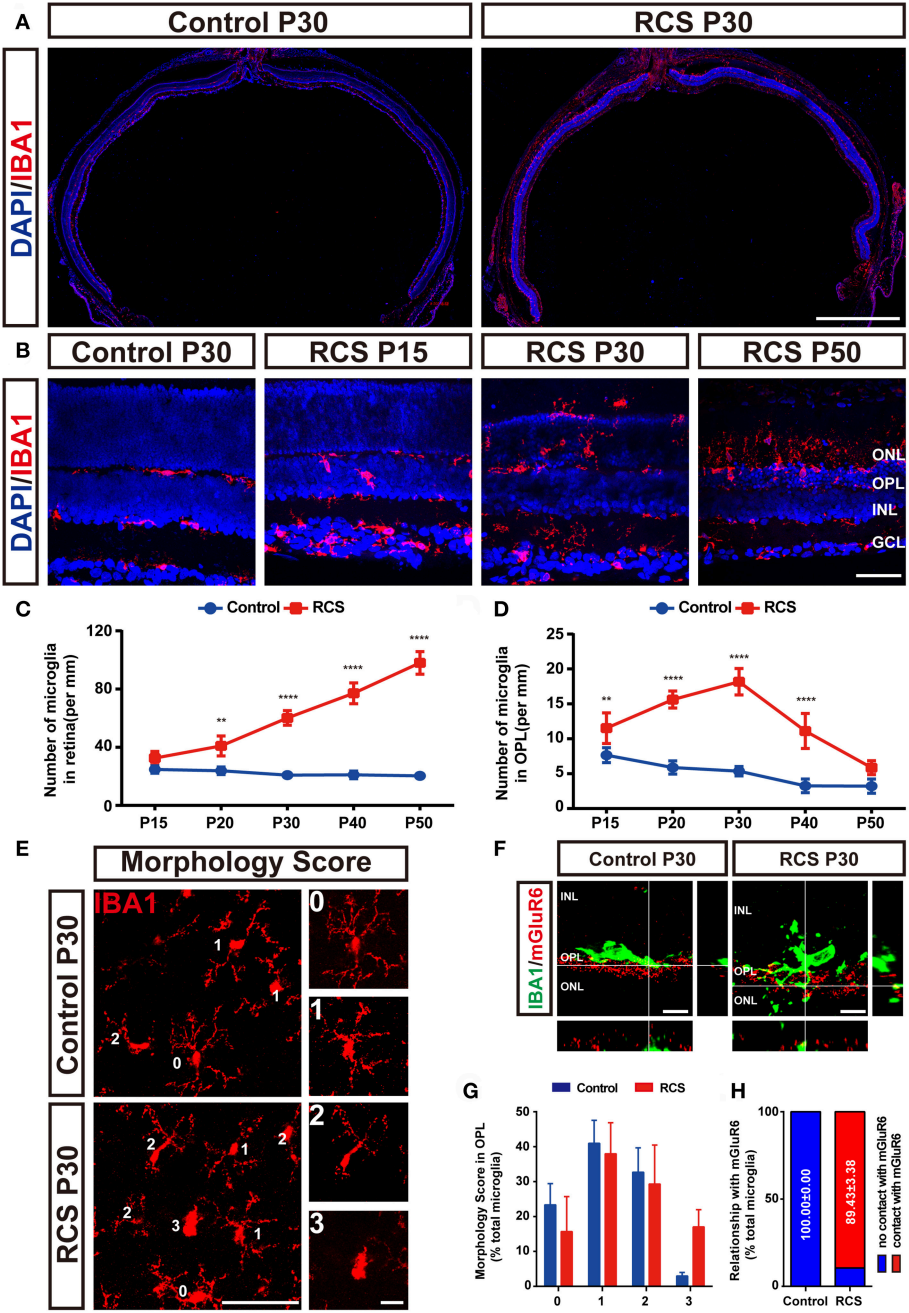
Increased number of microglia and their relationship with synaptic elements in the retinas of RCS rats. **(A)** Representative panoramic images of retinal sections stained with DAPI (blue) and IBA1 (red) from P30 control and RCS rats. **(B)** The distribution of microglia [IBA1 (red)] in the retinas of P30 control rats or P15, P30, and P50 RCS rats. **(C)** Quantification of the number of microglia in the total retinas indicated that the number of total retina microglia increased from P15 to P50 because of photoreceptor apoptosis (*N* = 3 eyes from different rats, *n* = 3 panoramic section images from each eye). **(D)** Quantification of the number of microglia in the OPL, indicating that the number of microglia in the OPL peaked at P30 (*N* = 3 eyes from different rats, *n* = 3 panoramic section images from each eye). **(E)** The morphology of the Iba1-positive cells in the OPL of flat-mounted retinas was examined. For morphology, a score of 0–3 points is shown in the right insets. **(F)** An orthogonal view of high-resolution confocal images suggested that microglia (green) processes extended into synapse lamination and contacted synaptic material (red) in the OPL of P30 RCS rats compared to controls. **(G)** No difference in morphological scores of microglia in the OPL was observed between RCS and control rats at P30. (*N* = 3 eyes from different rats, *n* = 5 flat-mounted images from each retina). **(H)** Almost all of microglia in the OPL contacted synaptic material in the RCS rats at P30. (*N* = 4 eyes from different rats, *n* = 10–15 microglia from each retina from each retina). ONL, outer nuclear layer; OPL, outer plexiform layer; INL, inner nuclear layer; IPL, inner plexiform layer; GCL, ganglion cell layer. Scale bar, 1 mm **(A)**, 50 μm (**B** and **E**) or 10 μm (**E** and **F**). Bars represent means; error bars represent SD. ^**^p < 0.01, ^****^p < 0.0001 using two-way ANOVA **(C,D,G)**.

Microglia play a key role in the phagocytic elimination of synaptic elements during the development or disease of the brain (12, 28, 36, 37). To explore their roles in the phagocytosis of synaptic elements, functional and morphological analysis of the microglia in the retinas of RCS rats were carried out by the immunofluence staining (Figures 4A–C and Supplementary Figures 2A,B). The number of retinal microglia in the OPL of the RCS rats markedly increased at P15 and peaked at P30 (Figure 4D), and synapse loss peaked at the same time point. The states of microglia were categorized by determining a morphological score. Appreciable differences in morphological scores of microglia in the OPL of RCS and control rats were not observed at P30 (Figures 4E,G), although most microglia showed an amoeboid morphology (a score of 2 or 3) in the ONL of RCS rats (Supplementary Figures 2C,D). In the mature normal retina, microglial processes were juxtaposed with presynaptic and postsynaptic structures in the OPL; however, microglial processes in the RCS OPL exhibited morphological alterations, extended into the synaptic layer and contacted mGluR6 (Figures 4F,H).

**Figure 5:**
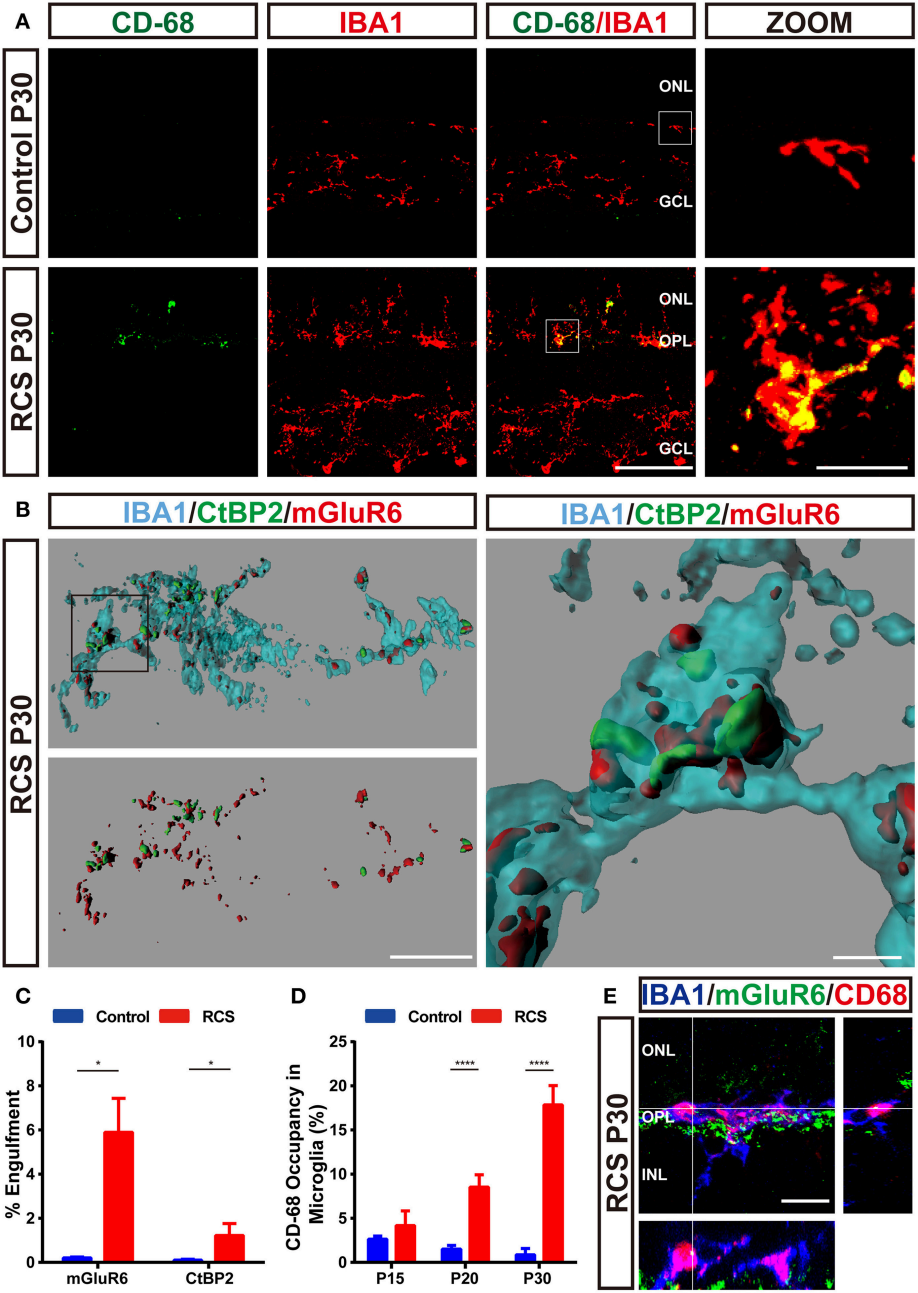
Engulfment of pre- and postsynaptic elements of RBCs by microglia in the retina of RCS rats. **(A)** Immunostaining for IBA1 (red) and CD-68 (green) in the retinas of P30 control and RCS rats suggested that retinal degeneration induced high levels of CD68 (green) immunoreactivity in Iba1-positive (red) microglia in the OPL. **(B,C)** Three-dimensional reconstruction and surface renderings prepared using Imaris revealed larger volumes of synaptic puncta inside microglia in the RCS OPL, particularly postsynaptic mGluR6 puncta, compared with controls (*N* = 3 eyes from different rats, *n* = 15–20 microglia from each retina). **(D)** Quantification of % volume of microglia occupied by CD68-positive lysosomes in the OPL of control and RCS rats at P15, P20, and P30 (*N* = 3 eyes from different rats, *n* = 10–15 microglia from each retina). **(E)** An orthogonal view of a representative high-resolution confocal image showed colocalization of mGluR6 (green) within the CD68-immunoreactive lysosomal compartment (red) of an Iba1-positive microglial cell (blue). ONL, outer nuclear layer; OPL, outer plexiform layer; INL, inner nuclear layer; IPL, inner plexiform layer; GCL, ganglion cell layer. Scale bar, 50 μm **(A)**, 10 μm **(A,B,E)** or 1 μm **(B)**. Bars represent means; error bars represent SD. ^*^p < 0.05; ^****^p < 0.0001 using an independent two-samples *t*-test **(C)** or two-way ANOVA **(D)**.

Microglia were double labeled with IBA1 and CD68 (Figure 5A and Supplementary Figures 2F–G), a marker of lysosomes specific to microglia, to clarify whether the microglia in the OPL phagocytosed synaptic elements in the RCS rats. The volume of microglia occupied by CD68-positive lysosomes exhibited a marked increase in the OPL of RCS rats at P20 and P30 compared to controls (Figure 5D). An *in vivo* phagocytosis assay (12, 28, 32) was used to confirm the engulfment of synaptic elements by phagocytosing microglia (Figure 5B). Rod degeneration resulted in a significantly higher volume of internalized postsynaptic mGluR6 and presynaptic CtBP2 in microglia in the OPL of RCS rats at P30 (Figure 5C), indicating that microglia phagocytosis of synaptic elements was increased when synapses were destroyed. An orthogonal view of a representative high-resolution confocal image showed colocalization of mGluR6 with the CD68-immunoreactive lysosomal compartment of Iba1-positive microglia (Figure 5E), suggesting that engulfed synapses were usually located in the internal lysosomal compartments of microglia.

### Depletion of Microglia Rescued the Postsynaptic mGluR6 Levels and Increased the Number and Length of Ectopic Dendrites in the RBCs

**Figure 6:**
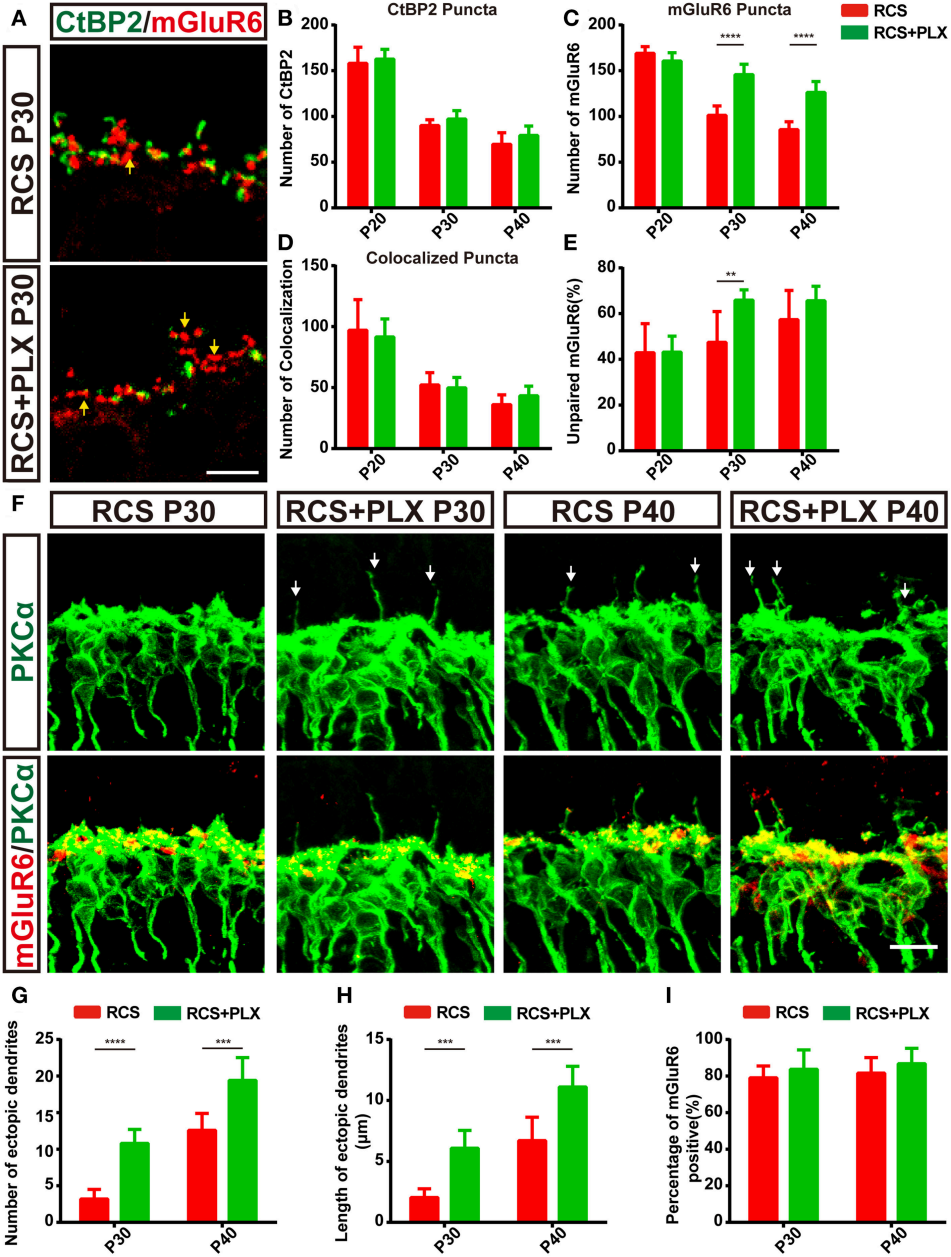
The elimination of microglia rescues the loss of postsynaptic mGluR6-positive elements and increases the number and length of ectopic RBC dendrites in RCS rats. **(A)** Confocal images of CtBP2- (green) and mGluR6-immunoreactive (red) puncta in the retinas of RCS rats and RCS rats treated with PLX3397 at P30. Yellow arrows indicate unpaired mGluR6-positive puncta. **(B–E)** Quantification of synaptic puncta or their apposition indicated that sustained microglia depletion reduced the loss of mGluR6-positive puncta and increased the proportion of mGluR6-positive puncta that were not paired with CtBP2-positive puncta; however, this depletion did not result in the alteration of colocalized puncta and presynaptic CtBP2-positive puncta (*N* = 3–5 eyes from different rats, *n* = 15–20 images from each eye). **(F)** Immunostaining for PKCα (green) and mGluR6 (red) in the retinas of RCS rats and RCS rats treated with PLX3397 at P30 and P40. White arrows indicate ectopic dendrites. **(G,H)** The number and length of ectopic RBC dendrites increased in the retinas of RCS rats and RCS rats treated with PLX3397 P30 and P40 in each field of view (213 × 213 μm) (*N* = 5 eyes from different rats, *n* = 7–12 images from each eye). **(I)** The proportion of mGluR6-positive ectopic RBC dendrites in each field of view (213 × 213 μm) of the retinas was not different between RCS rats and RCS rats treated with PLX3397 at P30 and P40 (*N* = 5 eyes from different rats, *n* = 7–12 images from each eye). ONL, outer nuclear layer; OPL, outer plexiform layer; INL, inner nuclear layer; GCL, ganglion cell layer. Scale bar, 5 μm **(A,F)**. Bars represent means; error bars represent SD. ^**^p < 0.01, ^***^p < 0.001, ^****^p < 0.0001 using two-way ANOVA **(B–E,G–I**).

To confirm the contribution of microglia to the clearance of synaptic material during retina degeneration in RCS rats, we eliminated microglia with the CSF1R inhibitor PLX3397 (600 ppm) (Supplementary Figures 3A–D), which was fed to 15-day-old RCS rats for 25 days (Supplementary Figure 3A). This compound was previously shown to efficiently deplete microglia in the CNS (38, 39). The number of microglia obviously reduced in both the OPL and the total retina 5 days after CSF1R administration and was maintained at low levels (Supplementary Figures 3B–D). Noticeable differences in the numbers of CtBP2-positive and colocalized puncta were not observed during microglia elimination in the RCS rats (Figures 6A,B,D); however, the number of mGluR6-positive puncta and the proportion of unpaired mGluR6-positive puncta increased significantly in the retinas of RCS rats after treatment with PLX3397 for 15 days (*p* < 0.01) (Figures 6A,C,E and Supplementary Figures 4A,B). Levels of the CtBP2 and mGluR6 mRNAs were not significantly altered between the depletion groups and untreated groups at P30 (Supplementary Figures 4E,F). Microglia depletion markedly increased the number of TUNEL-positive cells in the ONL of RCS rats (Supplementary Figures 3E,F and Supplementary Figure 4D); however, no significant changes in the number of surviving cells were observed between microglia-depleted retinas and untreated groups (Supplementary Figure 3G). Therefore, the microglia in the OPL of RCS rats mainly mediated the engulfment of postsynaptic elements.

In the RCS rat retina, ectopic dendrites emerged as early as P36 (27), the time at which the number of microglia in the OPL decreased, while the proportion of unpaired mGluR6 increased. In the present study, the RBC ectopic dendrites labeled with PKCα were observed as early as P30 in the RCS rats exhibiting sustained microglia depletion (Figure 6F). Significantly greater numbers and lengths of ectopic RBC dendrites were observed in the PLX3397-treated RCS rats than in RCS control rats (Figures 6G,H). However, the proportion of mGluR6-positive ectopic RBC dendrites (Figure 6I) was not significantly altered following the elimination of microglia from the RCS rats. Furthermore, in microglia-depleted retinas from control rats, abnormal outgrowth or retraction of dendritic and axonal compartments was not detected (Supplementary Figure 4C). Based on these findings, microglia elimination promoted ectopic neuritogenesis of RBCs in RCS rats.

### Influences of Microglia on the Dysfunction of Bipolar Cells in the RCS Retina

**Figure 7:**
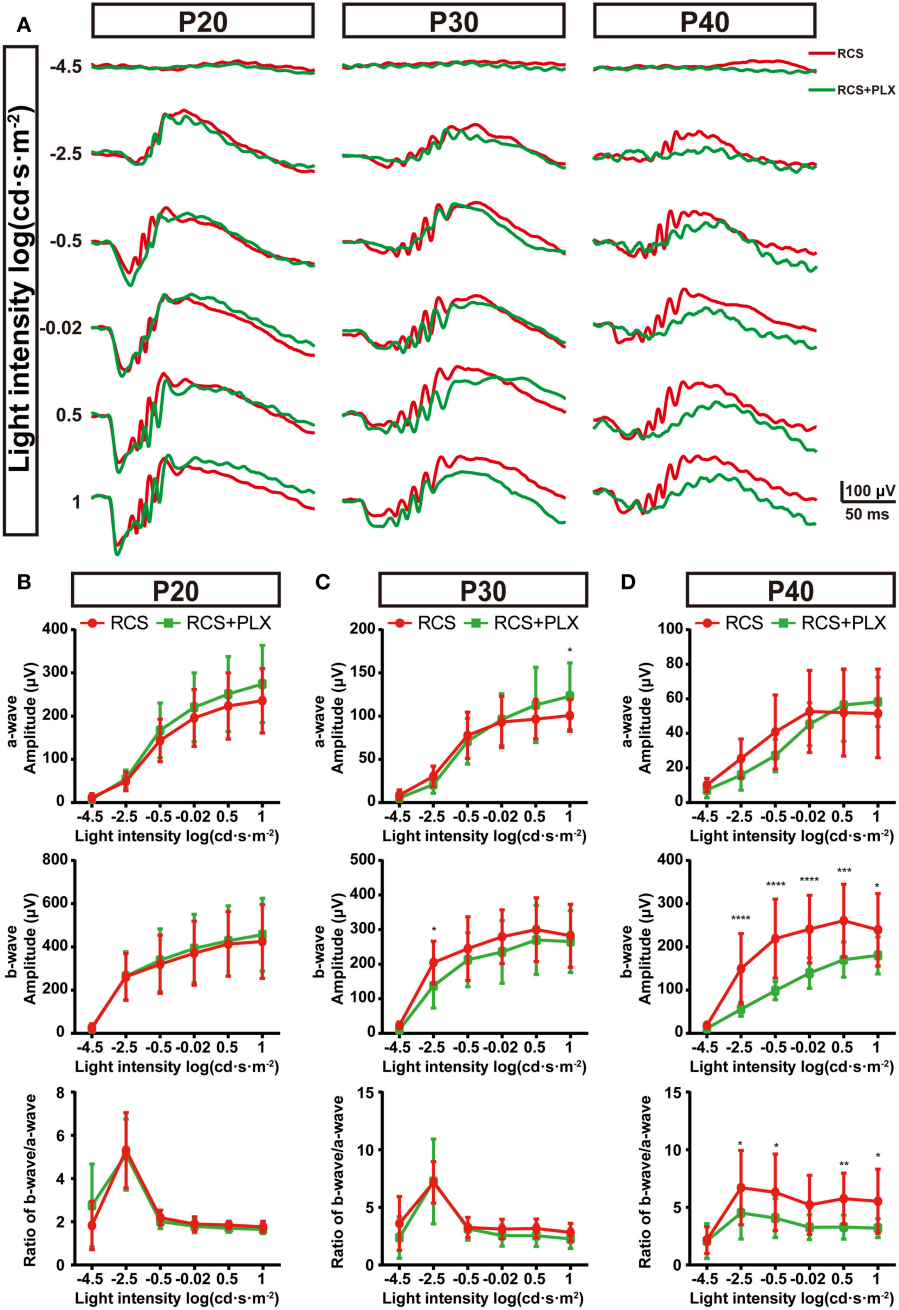
Elimination of microglia in the retinas of RCS rats reduces the amplitudes of ERG signals. **(A)** Representative light-evoked ERG waveforms measured with six different light intensities [from −4.5 to 1 log(cd·s·m^−2^)] in P20, P30, and P40 RCS rats and RCS rats treated with PLX3397. **(B–D)** Average stimulus-response curves for a-wave amplitudes (top row), b-wave amplitudes (middle row) and the ratio of a-wave/b-wave amplitudes in RCS rats and RCS rats treated with PLX3397 at P20 (*n* = 18 and 34, respectively), P30 (*n* = 16 and 28, respectively) and P40 (*n* = 15 and 16, respectively) show that sustained microglial depletion reduced the b-wave amplitude and did not affect the a-wave amplitude. This result was exacerbated over time. Bars represent means; error bars represent SD. ^*^p < 0.05; ^**^p < 0.01, ^***^p < 0.001, ^****^p < 0.0001 using two-way ANOVA **(B–D)**.

As structural damage of rod-bipolar synapse contributed to the visual dysfunction of RCS rats at the early stage, microglia probably mediated the visual dysfunction (5). To investigate this, we recorded ERG responses of RCS rats which were continuously depleted microglia for 5,15,25 d, and compared with time matched non-depleted RCS rats respectively (Figure 7A). Five days after microglial depletion, a-wave and b-wave amplitudes of scotopic ERG responses exhibited little change relative to untreated groups (*p* > 0.05) (Figures 7A,B). Fifteen days after the elimination of microglia, a modest decrease in a-wave amplitudes of scotopic ERG responses was only observed at the highest flash intensities, and a slight (but not significant) reduction in scotopic b-wave amplitudes was detected (Figures 7A,C). Twenty-five days after depletion, a-wave amplitudes of scotopic ERG responses showed no significant changes, whereas b-wave amplitudes showed a pronounced decrease (*p* < 0.05) (Figures 7A,D). Thus, the scotopic b-wave response, originating from postsynaptic transmission from the photoreceptors to RBCs, progressively decreased in the RCS rats. As a result, the b–a amplitude ratios decreased as microglial depletion progressed (Figure 7D). The control rats that received PLX3397 for 5, 15, and 25 days did not show any significant changes in a- or b-wave amplitudes under dark-adapted conditions (Supplementary Figure 5), indicating that the observed ERG changes were mainly attributed to microglial depletion in the RCS rats, rather than to PLX3397 *per se*.

### Microglia Engulfed Postsynaptic Elements in a Complement-Dependent Mechanism

**Figure 8:**
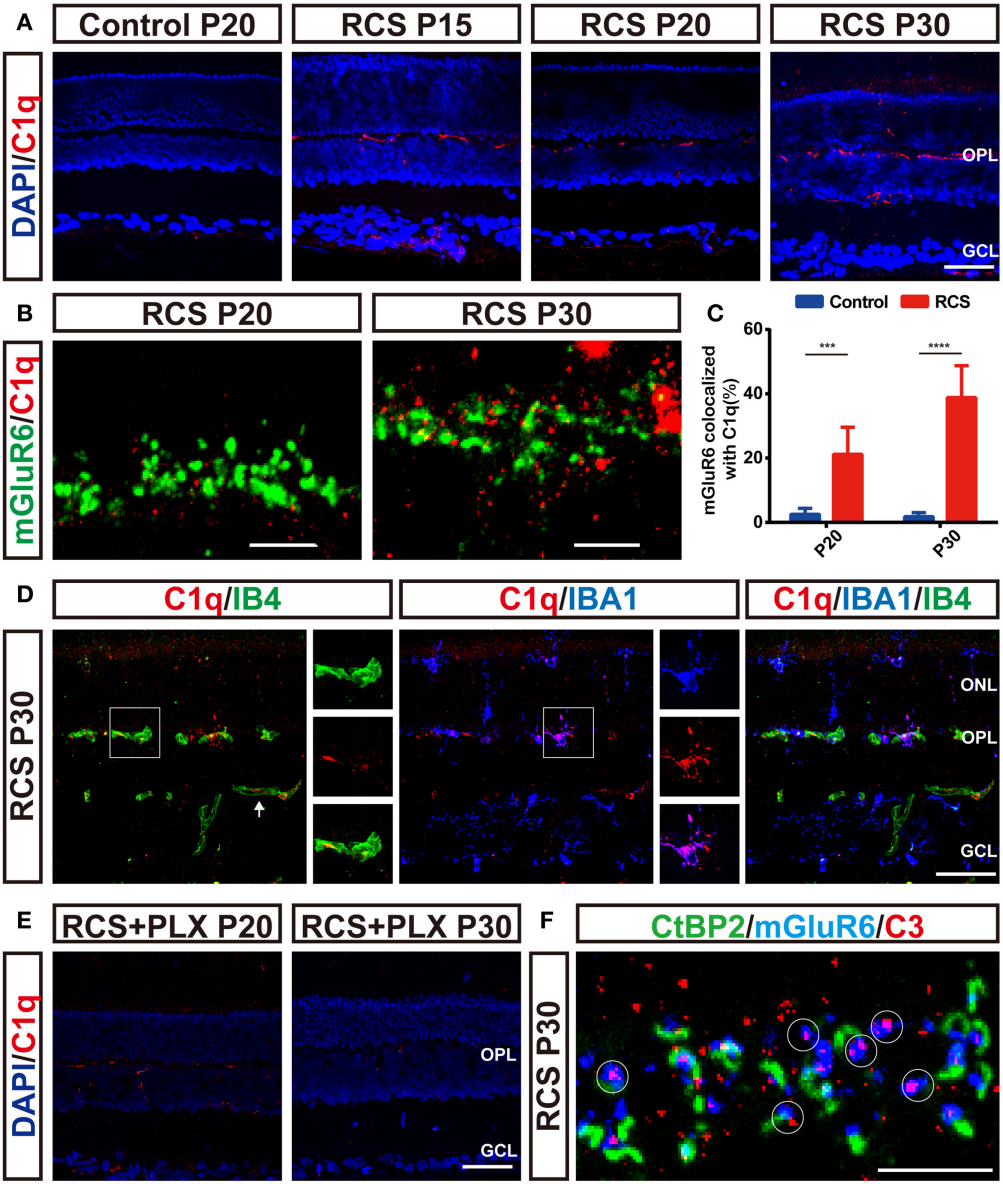
Microglia-induced C1q upregulation and deposition onto postsynaptic elements precede synapse loss in the OPL during retinal degeneration in RCS rats. **(A)** Immunostaining for C1q (red) in the retinas revealed region-specific (OPL) upregulation of C1q (red) in RCS rats. **(B,C)** Confocal images showing an increase in the colocalization of C1q (red) and mGluR6 (green) in RCS rats compared with controls (*N* = 6 eyes from different rats, *n* = 6–10 images from each eye). **(D)** Labeling of the retinal vasculature using isolectin-B4 (IB4) and microglia using IBA1 revealed that C1q in the OPL was mainly derived from the blood circulation and microglia in the P30 RCS rats. White arrows indicated that C1q came from the vasculature in the inner retina. **(E)** Elimination of microglia with the PLX3397 treatment decreased C1q immunoreactivity in the retinas of RCS rats. **(F)** Representative confocal image showing the colocalization of C3 (red) and mGluR6 (blue) but a lack of colocalization with CtBP2 (green) in the retinas of P30 RCS rats. White circles indicate C3 deposition onto unpaired mGluR6. ONL, outer nuclear layer; OPL, outer plexiform layer; INL, inner nuclear layer; GCL, ganglion cell layer. Scale bar, 50 μm **(A,D,E)**, 5 μm **(B,F)**. Bars represent means; error bars represent SD. ^***^p < 0.001, ^****^p < 0.0001 using two-way ANOVA **(C)**.

As the complement pathway mainly governs microglia-mediated engulfment of endogenous materials (40–42), we examined the distribution of C1q and C3 in the RP retina in the present study. C1q immunoreactivity was elevated in RCS retinas as early as P15 and preceded synapse loss (Figure 8A). Notably, the C1q level was increased in a region-specific manner, particularly in the OPL, the region susceptible to synapse loss. Confocal images confirmed the colocalization of C1q and mGluR6 puncta in the RCS OPL (Figure 8B), and a higher percentage of mGluR6-positive puncta colocalized with C1q-positive puncta in the OPL of RCS rats at P20 and P30 than in the control groups (Figure 8C). Thus, C1q mediated the engulfment of mGluR6-positive postsynaptic elements. Furthermore, double staining for C1q and IB4 or IBA1 revealed that C1q was mainly localized in the vasculature and microglia in the retina of RCS rats (Figure 8D). Sustained microglial depletion significantly decreased C1q levels in the retinas of RCS rats (Figure 8E). C3, which is activated by C1q to opsonize synapse elimination (28, 42–44), was also deposited on the unpaired mGluR6-positive puncta in the OPL of RCS rats at P30 (Figure 8F). Therefore, microglia might phagocytize postsynaptic elements through a complement-dependent manner.

## Discussion

**Figure 9:**
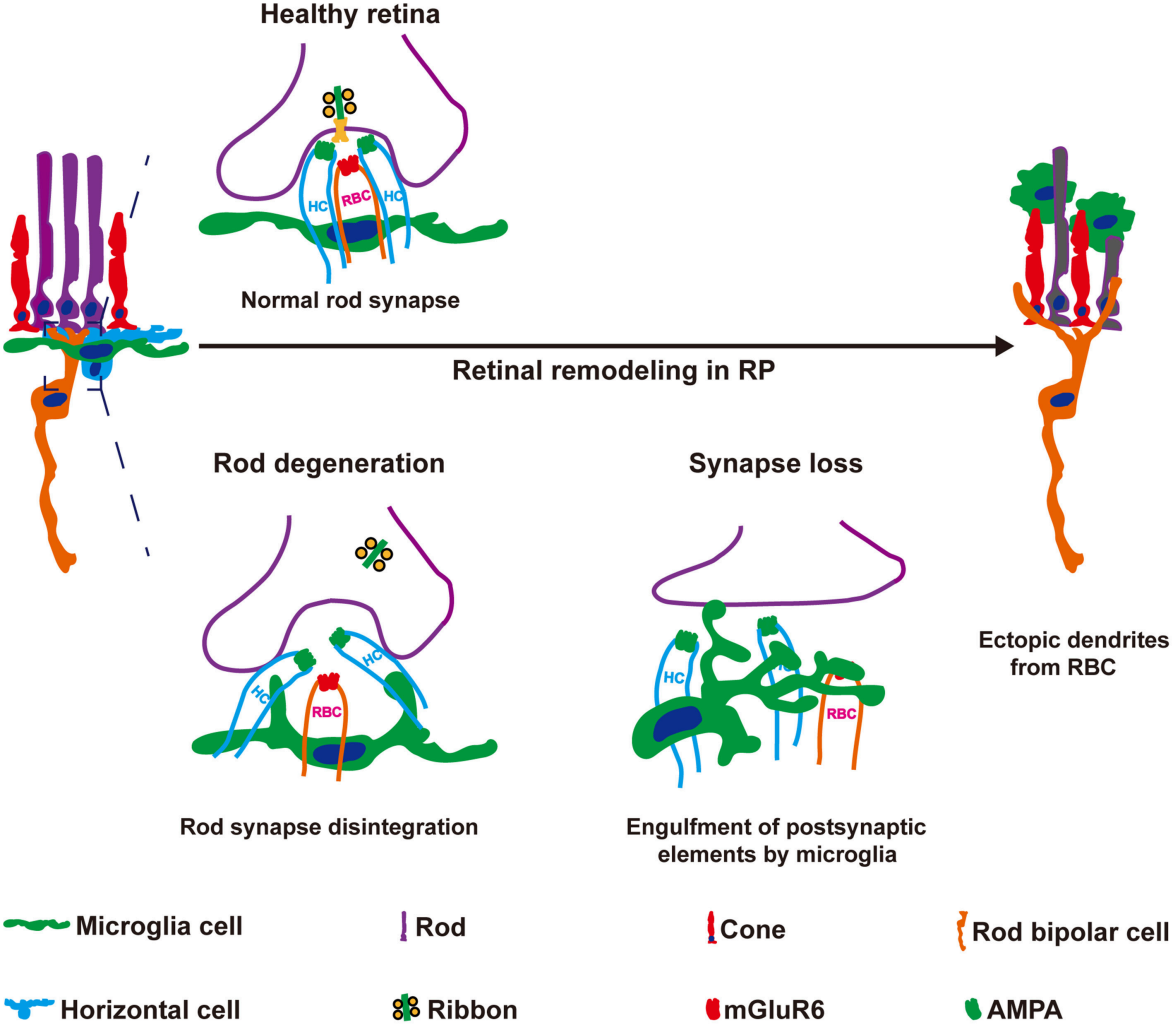
Microglia engulf postsynaptic elements and mediate the formation of ectopic dendrites in the retinas of RCS rats. Synaptic remodeling of neuronal circuits in the OPL are early hallmarks of RP that are thought to be initiated by photoreceptor degeneration. However, the mechanisms underlying synapse loss and remodeling remain elusive. Here, microglia-mediated engulfment of postsynaptic elements was a mechanism underlying ectopic RBC neuritogenesis. Rod degeneration perturbed RBC dendrite invagination into the photoreceptor terminus, and then these postsynaptic elements of RBCs were engulfed by microglia. Inhibition of the microglial engulfment of synaptic material using a pharmacological method increased postsynaptic mGluR6 expression and the formation of ectopic RBC dendrites, but decreased the ERG responses in RCS rats. Based on these findings, microglial cells play vital roles in the synapse loss and remodeling observed in RCS rats.

In the present study, we observed novel roles for microglial in the early synaptic remodeling of neuronal circuits during retinal degeneration. Resident microglia in the retina OPL phagocytosed postsynaptic elements of RBCs at the early stage of RCS rats (Figure 9). Sustained microglial depletion by PLX3397 blocked synapse loss, increased ectopic neuritogenesis of RBCs and further exacerbated the deterioration of visual function. C1q expression was induced by microglia, and classical complement pathway were revealed involvement in clearance of postsynaptic elements. Based on our data, microglia mediated the remodeling of neuronal circuits in RBCs, contributing to the maintenance of vision at the early stage of RP.

During retinal degeneration, changes in synapse morphology were detected at P21 (5). In the present study, we confirmed that rod-RBC synapses were lost in the OPL of RCS rats as early as P20 and reached a value of 50% at P30. Notably, floating ribbons and RBC dendritic tips extending from rod spherules were observed using electron microscopy, and some of these abnormal synaptic contacts between rods and RBCs were called the flat-contact type (34). These results implied that the loss of presynaptic and postsynaptic elements in the OPL did not occur through the simultaneous degradation of both elements in intact synapses. Changes in the size of the presynaptic specialization of cone synapses occurred earlier than changes in the size of the postsynaptic density, indicating that photoreceptor degeneration induced sequential presynaptic protein loss, synaptic disintegration and eventually postsynaptic element loss. In some retinal degeneration models, synapses were reported to form normally (6, 24, 45). Thus, photoreceptor synaptogenesis could occur before photoreceptor degeneration. In RCS rats, progressive photoreceptor degeneration was detected after the time of eye opening (at P15 in rats) (25, 34, 46). In the present study, synapse levels in the retinas of RCS rats were not altered at P15 compared with control rats. Our data from transmission electron microscopy further confirmed normal synaptic structures in P15 RCS rat retinas. Based on our data, synaptic element loss was likely not a result of abnormal synaptic development, but a consequence of photoreceptor degeneration.

Coincident with RBC dendritic retraction from the OPL after rod degeneration, mGluR6 disappeared from RBC dendrites (7, 8), raising the possibility that mGluR6 loss was a consequence of a rapid and local disassembly of ON-bipolar cell dendrites (47, 48). However, deletion of RIBEYE, which substantially decreases the number of ribbons in retinal synapses and disrupts neurotransmitter release, does not affect the overall organization and synaptic connectivity of the retina (49). The loss of the typical triad organization does not disturb the gross structure of bipolar dendritic tips, including mGluR6 accumulation (50–52). Together with findings from the present report, perturbations in synaptic inputs and integrity did not directly lead to mGluR6 elimination via degradation, suggesting that synapse loss might have resulted from phagocytosis by microglia cells. The presynaptic materials, including ribbons, disappeared possibly as a part of the mechanism by which phagocytic microglia remove rod cells in the ONL (16) or were decomposed through a lysosomal-mediated autophagic process at the early stage of rod apoptosis (53).

Microglia are known to play a role in the pruning of synapses during CNS development and neurodegeneration (54). Microglia play key roles in the selective clearance of presynaptic debris during Wallerian degeneration (36) or the elimination of postsynaptic elements in subjects with Alzheimer's disease (AD) (28). In the present study, microglia in the OPL mainly engulfed postsynaptic mGluR6, with little effect on synaptic integrity or presynaptic ribbon loss. Following the formation of direct contacts, the OPL microglia engulfed postsynaptic debris, translocating it to phagosomes for destruction. Similar to subjects with AD (28), microglia in the RCS OPL exhibited the similar ramified morphology as controls, indicating that photoreceptor stress induced synapse engulfment by resident homeostatic microglia. Synapse disintegration was not blocked by microglia depletion in RCS rats, suggesting that the disruption of the synaptic triad pattern was not initiated by phagocytosing microglia.

In RCS rats, the microglia in the retinas also carried the MERTK mutation. Our present data showed that Mertk mutation did not influence the synapses phagocytosis of microglia in the OPL of RCS rats at P30. However, more apoptotic cells were observed in the ONL of the RCS rats after microglia were deleted by PLX3397 treatment. It seemed that Mertk-deficient did not interrupt the phagocytosis of the microglia completely and the microglia kept the ability to engulf apoptotic cells and synaptic elements. To date, the best characterized mediators of synaptic pruning are components of the complement cascade (12). Previous experiments implicated the complement system as a key driver of the clearance of neuronal materials by microglia during development (12, 55, 56), and in subjects with AD (28), schizophrenia (57), viral infection (58), and some types of retinal degeneration (59, 60). While most of these latter studies had not shown interest in microglia-mediated removal of synapses via complement, we speculated that this pathway would be confirmed to promote synaptic elimination and that inhibition of this pathway or its components would prevent engulfment by microglia. Moreover, local activation of the complement pathway might serve as a key mechanism underlying rod apoptosis-induced synapse pruning in the RP retina. C1q was not only produced by microglia but was also derived from the circulatory system in RP retinas. Microglia depletion in RCS rats significantly ameliorated C1q deposition, suggesting that microglia played a crucial role in activating the complement cascade and driving synapse elimination.

In a recent study, microglial depletion using transgenic mice perturbed synaptic function and integrity in the mature retina (44); however, synapse formation and ERG responses in control rat retinas were not changed by pharmacological elimination of microglia in this study. A similar phenomenon was observed in the adult brain, as mice depleted of microglia showed deficits in multiple learning tasks and a significant reduction in motor-learning-dependent synapse formation using the same genetic methods of microglial depletion (61) as in the mature retina (44). However, behavioral or cognitive abnormalities were not detected in the mice treated with PLX3397 (39), suggesting that the discrepancies in the functional conclusions of sustained microglia elimination were due to the use of different experimental methods. Furthermore, tamoxifen, which is used to manipulate genetic microglia elimination, exerts protective effects on retinal degeneration (62). Thus, pharmacological depletion of microglia by a CSF1R inhibitor was a more suitable approach to explore the function of microglia.

In our study, the b-wave amplitude in the ERG, which reflected RBC function, was progressively reduced during microglial depletion in the RCS retina. However, significant changes in surviving photoreceptors and a-wave amplitudes were not observed in RCS rats treated with PLX3397, suggesting that sustained microglia depletion did not affect the function of photoreceptors in the RP rat model. The same phenomenon was observed in a model of acute injury of optic nerves, in which retinal ganglion cell (RGC) degeneration remained unaffected upon microglia depletion by PLX5622 (another CSF1R inhibitor) (63). Based on our results, sustained microglial depletion aggravated the response of RBCs to light in RCS rats. Thus, the abnormal accumulation of mGluR6 in RBCs damaged their function, providing further evidence that the engulfment of postsynaptic elements by microglia in the OPL of RCS rats improved the light response of RBCs.

According to a recent report from our group, miR-125b-5p regulates the formation of ectopic dendrites from RBCs into the ONL and functional synapse formation with the remaining photoreceptors in RCS rats (27). However, the key mechanism triggering ectopic neuritogenesis remains unknown. Ectopic dendrites of RBCs might form to restore their input activity in the degenerating retina (10, 27, 64, 65), suggesting that the dysfunction of RBCs might be a key trigger of remodeling (10). Based on our data that depletion of microglia promoted ectopic neuritogenesis, microglia were shown to be involved in the mechanism regulating ectopic dendrite formation by the RBCs of RCS rats. We also hypothesized that microglia subsequently delayed ectopic neuritogenesis by restoring function of RBCs.

In conclusion, microglial cells did not only contribute to the survival of photoreceptors and to the maintenance of retinal architecture (16, 18, 19), but also mediated synaptic remodeling of neuronal circuits by phagocytosing synapses in the RP retina. In our study, a CSF1R inhibitor exerted detrimental effects on the visual function of a RP rat model, suggesting that this type of drug-induced non-selective microglial elimination was not suitable to restore the vision of patients with RP. A treatment that selectively stimulates synaptic phagocytosis by microglia in the retina would be an important topic in future studies of retinal degenerative diseases. Based on our findings, novel treatment strategies targeting microglia should add mechanisms governing microglia–synapse interactions, and not simply aim to support photoreceptor survival.

## Ethics Statement

This study was carried out in accordance with the recommendations of Third Military Medical University Animal Care and Use Committee. The protocol was approved by the Animal Center of the Third Military Medical University.

## Author Contributions

JH: conception and design, collection and assembly of data, data analysis and interpretation, manuscript writing. JD, LG, YF, CW, BB, HL, and YG: collection and assembly of data. CZ: conception and design. HX: conception and design, data analysis and interpretation, manuscript writing, final approval of manuscript. ZY: conception and design, financial support, data analysis and interpretation, final approval of manuscript.

### Conflict of Interest Statement

The authors declare that the research was conducted in the absence of any commercial or financial relationships that could be construed as a potential conflict of interest.

## Supplementary Material

Supplementary Figure 1 The change of synapse in the OPL during retinal degeneration in RCS rats. **(A)** Schedule of synapse analysis in the OPL at the early stage of retinal degeneration in RCS rats. **(B,C)** Confocal images of CtBP2- (green) and mGluR6-immunoreactive (red) puncta in the retinas of control and RCS rats at P15 and P20. **(D,E)** CtBP2 and mGluR6 mRNA expression in the retinas of control and RCS rats at P15, P20, P30, P40, and P50 (*N* = 3 rats per group). **(F,G)** Immunostaining for CtBP2 (green), mGluR6 (red), and cone arrestin (blue) in the retinas of control and RCS rats at P15 and P20 respectively. ONL, outer nuclear layer; OPL, outer plexiform layer; IPL, inner plexiform layer; Scale bar, 50 μm **(B,C)** or 5μm **(F,G)**. Bars represent means; error bars represent SD. ^**^*p* < 0.01, ^****^*p* < 0.0001 using two-way ANOVA **(D,E)**.

Supplementary Figure 2 Microglia distribution and morphology in the retina of control and RCS rats. **(A)** Representative panoramic images of retinal sections stained with DAPI (blue) and IBA1 (red) from P15 and P20 RCS rats. **(B)** Representative panoramic images of retinal sections stained with DAPI (blue) and IBA1 (red) from P40 and P50 RCS rats. **(C)** Representative confocal image showing the morphology of microglia (red) in the ONL of RCS rats at P30. **(D)** Percentages of microglia displaying each score in the RCS ONL. **(E)** An orthogonal view of a representative high-resolution confocal image showed the relationship between mGluR6 (green), CD68 (red) and Iba1-positive microglial cell (blue). **(F,G)** Immunostaining for IBA1 (red) and CD-68 (green) in the retinas of P15 and P20 RCS rats. ONL, outer nuclear layer; OPL, outer plexiform layer; IPL, inner plexiform layer; Scale bar, 1 mm **(A,B)**, 50 μm **(C,E,F)**. Bars represent means; error bars represent SD.

Supplementary Figure 3 Effects of microglia elimination on the apoptosis and survival of photoreceptors in the retinas of RCS rats. **(A)** Schematic of microglial elimination and time points examined in the RCS rats. **(B)** Panoramic images of retinal sections from PLX-administered RCS rats immunostained with Iba1 showed the presence and density of retinal microglia. **(C,D)** Quantification of the total number of retinal microglia and number of OPL microglia. Although microglial numbers were substantially increased in RCS rats, PLX-treated RCS rats exhibited sustained microglial elimination (>90%) in the OPL for up to 25 days. **(E)** Representative high-resolution confocal images showing photoreceptor apoptosis using TUNEL (green) in the retinas of RCS rats and RCS rats treated with PLX. **(F,G)** Quantification of DAPI- and TUNEL-positive photoreceptor cells in the ONL at three time points did not reveal significant differences in the number of surviving cells between RCS rats and RCS rats treated with PLX, although the number of TUNEL-positive photoreceptor cells was substantially increased following sustained microglia depletion. ONL, outer nuclear layer; OPL, outer plexiform layer; INL, inner nuclear layer; IPL, inner plexiform layer; GCL, ganglion cell layer. Scale bar, 50 μm **(B,E)**. Bars represent means; error bars represent SD. ^*^*p* < 0.05; ^***^*p* < 0.001, ^****^*p* < 0.0001 using two-way ANOVA **(C,D,F,G)**.

Supplementary Figure 4 The influence of microglia elimination on the synapses in RCS rats or photoreceptors in control rats. **(A,B)** Confocal images of CtBP2- (green) and mGluR6-immunoreactive (red) puncta in the retinas of RCS rats and RCS rats treated with PLX3397 at P20 and P40. **(C)** Immunostaining for CtBP2 (green), PKCα (red), and mGluR6 (blue) in the retinas of control rats treated with PLX3397 at P40. **(D)** Representative high-resolution confocal images showing photoreceptor apoptosis using TUNEL (green) in the retinas of control rats and control rats treated with PLX3397 at P40. **(E,F)** An analysis CtBP2 and mGluR6 mRNA expression in the retinas of control rats, control rats treated with PLX, RCS rats, and RCS rats treated with PLX at P30 suggested that PLX had no effect on CtBP2 and mGluR6 expression (*N* = 3 rats per group). ONL, outer nuclear layer; OPL, outer plexiform layer; INL, inner nuclear layer; IPL, inner plexiform layer; GCL, ganglion cell layer. Scale bar, 50 μm **(A-D)**. Bars represent means; error bars represent SD. using two-way ANOVA **(E,F)**.

Supplementary Figure 5 Effects of microglia eliminate on the amplitudes of ERG signals in control rats. **(A)** Representative light-evoked ERG waveforms measured with six different light intensities [from -4.5 to 1 log(cd.s.m-2)] in P20, P30, and P40 control rats and control rats treated with PLX3397. **(B-D)** Average stimulus-response curves for a-wave amplitudes (top row), b-wave amplitudes (middle row) and the ratio of a-wave/b-wave amplitudes in control rats and control rats treated with PLX3397 at P20 (*n* = 14 and 8 rats, respectively), P30 (*n* = 20 and 10 rats, respectively), and P40 (*n* = 6 and 6 rats, respectively). Bars represent means; error bars represent SD. using two-way ANOVA **(B-D)**.
