# Supplementary figures and images for: Microglia Mediate Synaptic Material Clearance at the Early Stage of Rats With Retinitis Pigmentosa
Source: Front Immunol. 2019 Apr 26;10:912. doi: 10.3389/fimmu.2019.00912 (PMC6499027; doi:10.3389/fimmu.2019.00912)

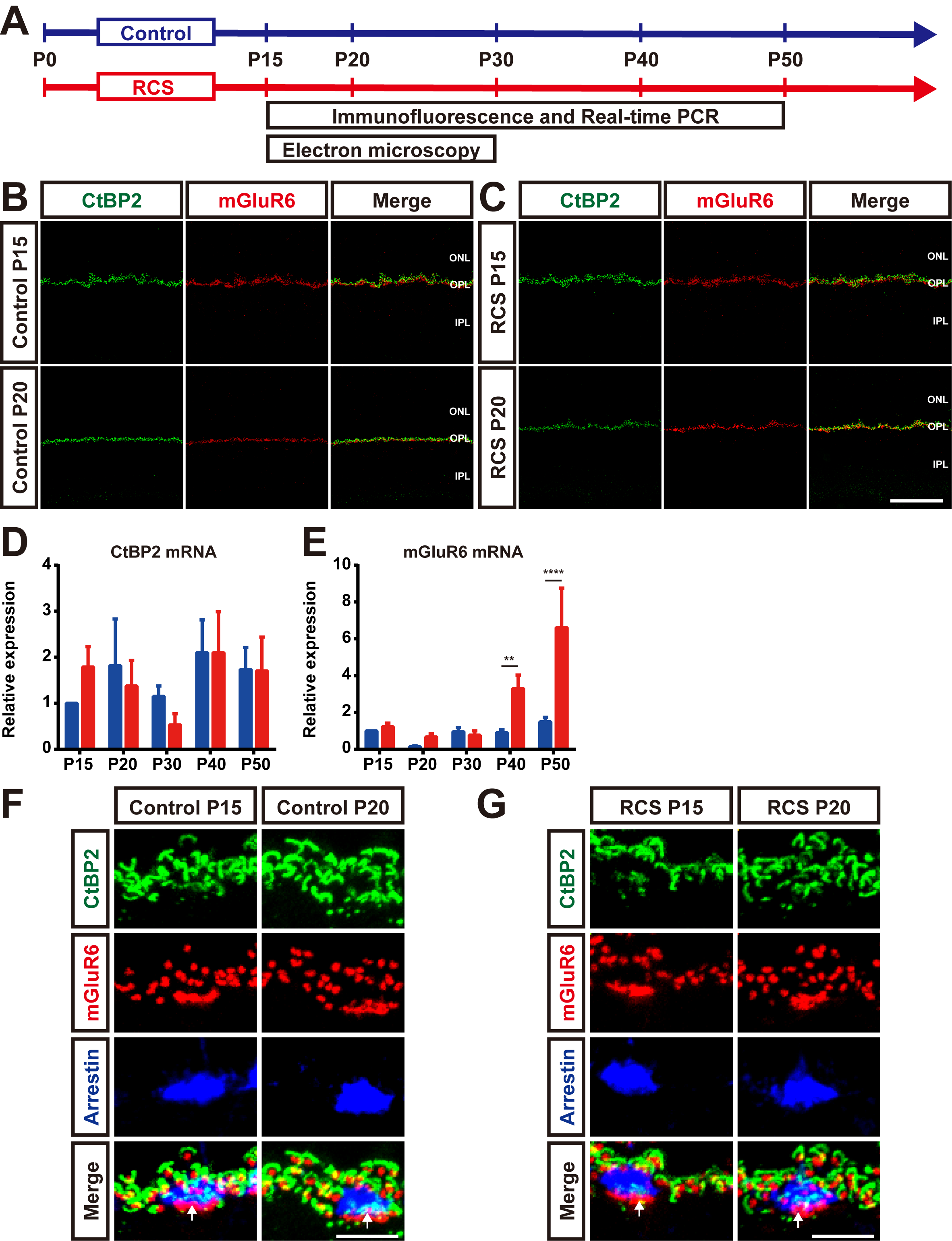

Supplement: Supplementary Figure 1 — The change of synapse in the OPL during retinal degeneration in RCS rats. (A) Schedule of synapse analysis in the OPL at the early stage of retinal degeneration in RCS rats. (B,C) Confocal images of CtBP2- (green) and mGluR6-immunoreactive (red) puncta in the retinas of control and RCS rats at P15 and P20. (D,E) CtBP2 and mGluR6 mRNA expression in the retinas of control and RCS rats at P15, P20, P30, P40, and P50 (N = 3 rats per group). (F,G) Immunostaining for CtBP2 (green), mGluR6 (red), and cone arrestin (blue) in the retinas of control and RCS rats at P15 and P20 respectively. ONL, outer nuclear layer; OPL, outer plexiform layer; IPL, inner plexiform layer; Scale bar, 50 μm (B,C) or 5μm (F,G). Bars represent means; error bars represent SD. **p < 0.01, ****p < 0.0001 using two-way ANOVA (D,E). [file Image_1.TIF]

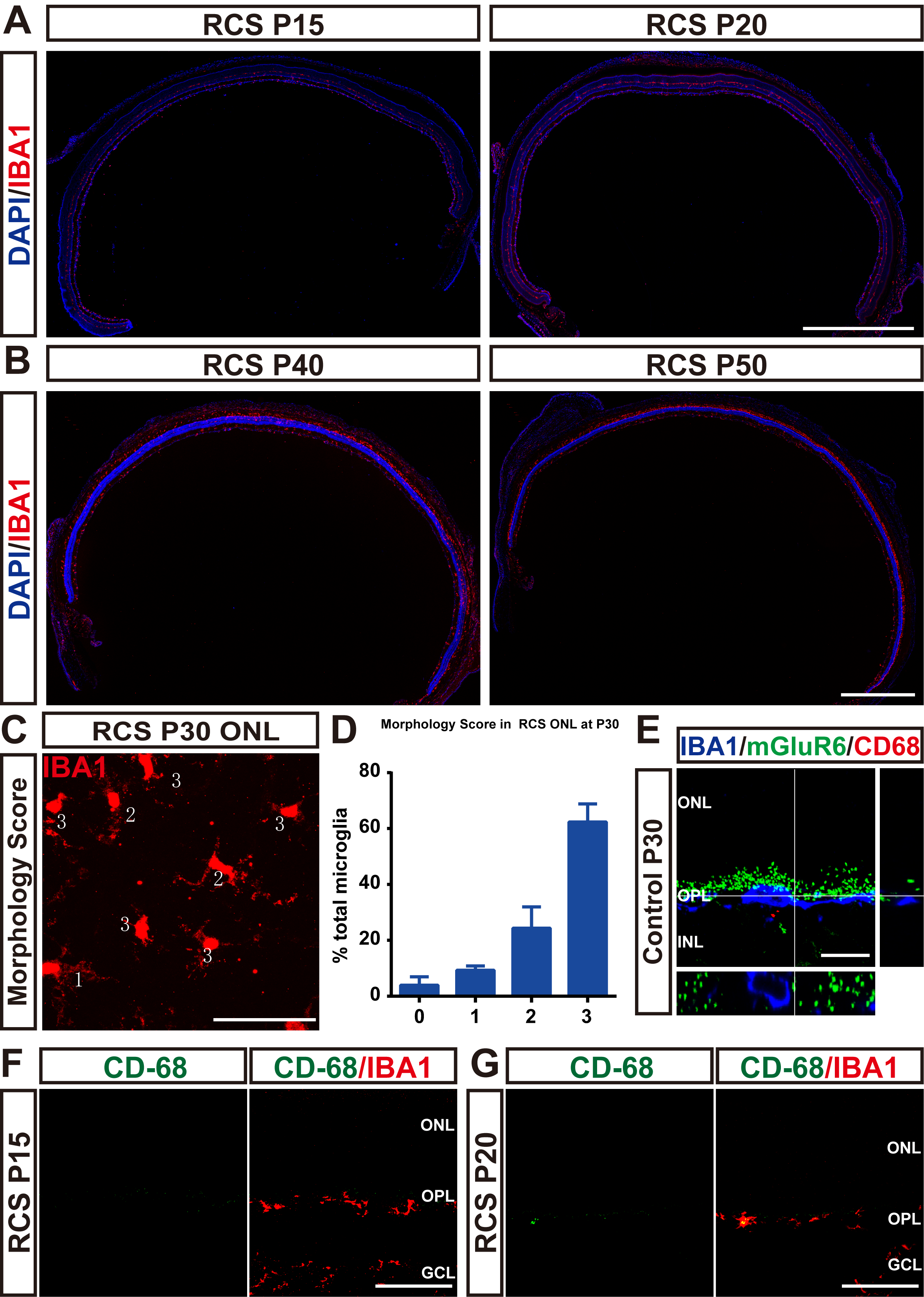

Supplement: Supplementary Figure 2 — Microglia distribution and morphology in the retina of control and RCS rats. (A) Representative panoramic images of retinal sections stained with DAPI (blue) and IBA1 (red) from P15 and P20 RCS rats. (B) Representative panoramic images of retinal sections stained with DAPI (blue) and IBA1 (red) from P40 and P50 RCS rats. (C) Representative confocal image showing the morphology of microglia (red) in the ONL of RCS rats at P30. (D) Percentages of microglia displaying each score in the RCS ONL. (E) An orthogonal view of a representative high-resolution confocal image showed the relationship between mGluR6 (green), CD68 (red) and Iba1-positive microglial cell (blue). (F,G) Immunostaining for IBA1 (red) and CD-68 (green) in the retinas of P15 and P20 RCS rats. ONL, outer nuclear layer; OPL, outer plexiform layer; IPL, inner plexiform layer; Scale bar, 1 mm (A,B), 50 μm (C,E,F). Bars represent means; error bars represent SD. [file Image_2.TIF]

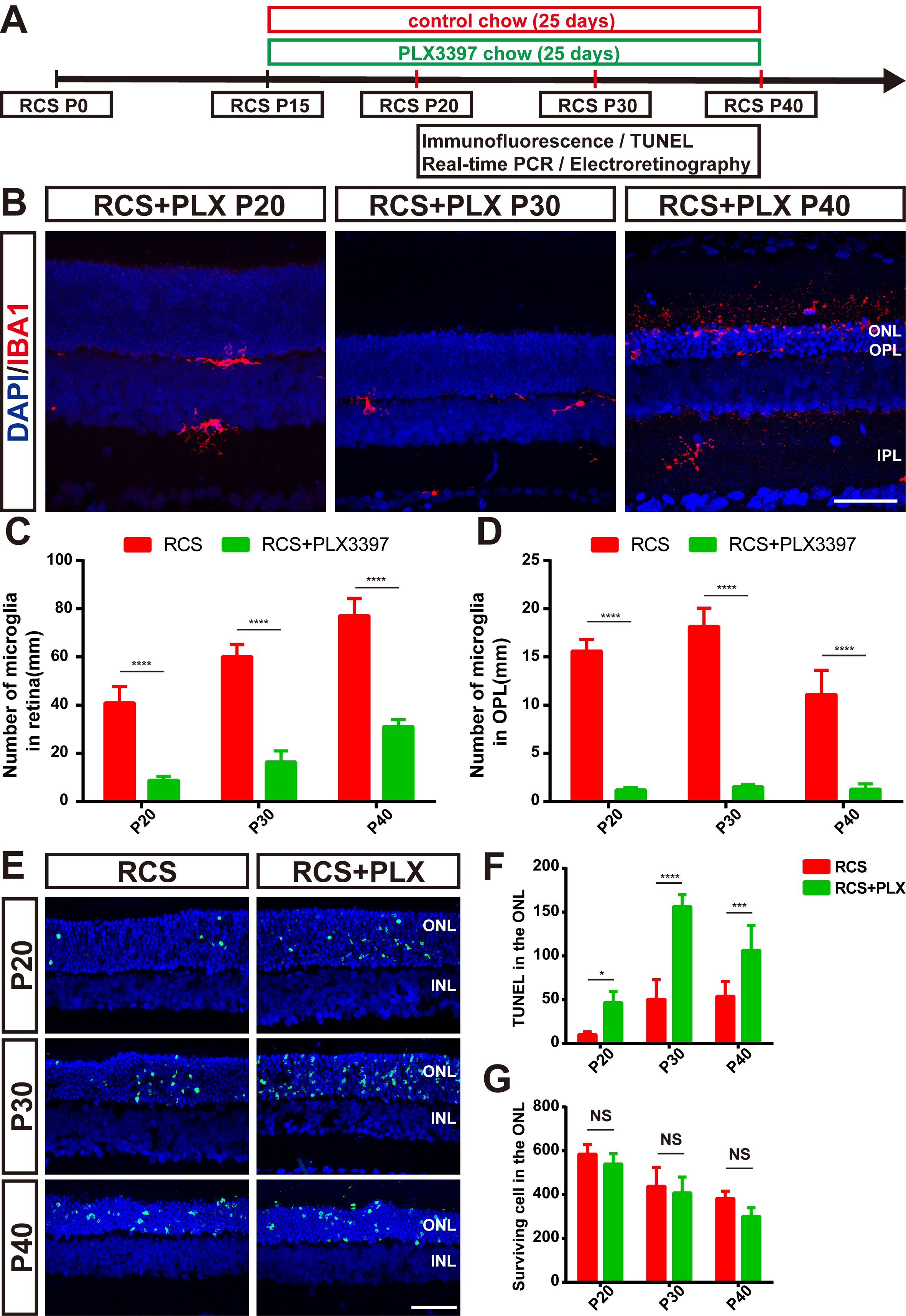

Supplement: Supplementary Figure 3 — Effects of microglia elimination on the apoptosis and survival of photoreceptors in the retinas of RCS rats. (A) Schematic of microglial elimination and time points examined in the RCS rats. (B) Panoramic images of retinal sections from PLX-administered RCS rats immunostained with Iba1 showed the presence and density of retinal microglia. (C,D) Quantification of the total number of retinal microglia and number of OPL microglia. Although microglial numbers were substantially increased in RCS rats, PLX-treated RCS rats exhibited sustained microglial elimination (>90%) in the OPL for up to 25 days. (E) Representative high-resolution confocal images showing photoreceptor apoptosis using TUNEL (green) in the retinas of RCS rats and RCS rats treated with PLX. (F,G) Quantification of DAPI- and TUNEL-positive photoreceptor cells in the ONL at three time points did not reveal significant differences in the number of surviving cells between RCS rats and RCS rats treated with PLX, although the number of TUNEL-positive photoreceptor cells was substantially increased following sustained microglia depletion. ONL, outer nuclear layer; OPL, outer plexiform layer; INL, inner nuclear layer; IPL, inner plexiform layer; GCL, ganglion cell layer. Scale bar, 50 μm (B,E). Bars represent means; error bars represent SD. *p < 0.05; ***p < 0.001, ****p < 0.0001 using two-way ANOVA (C,D,F,G). [file Image_3.TIF]

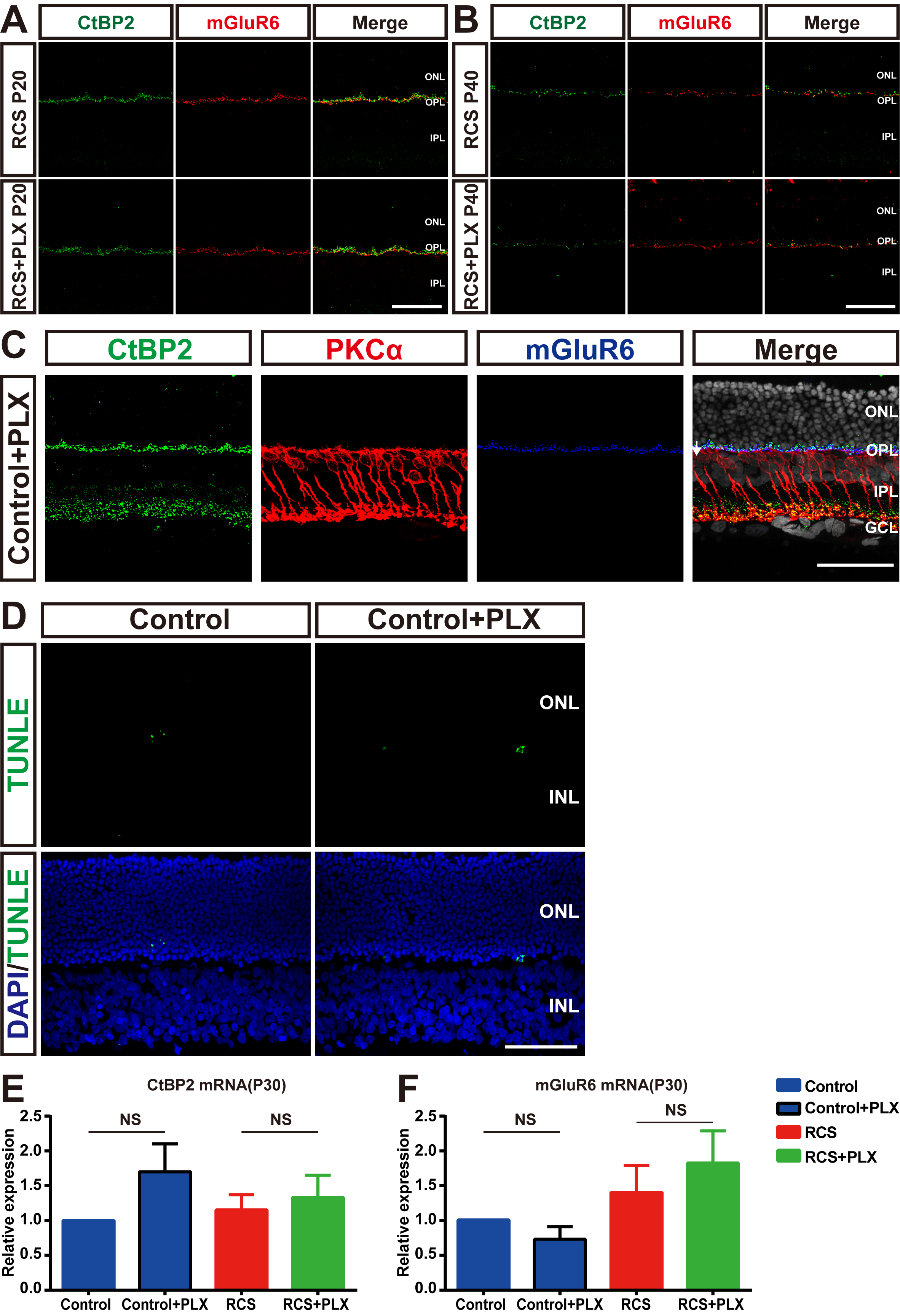

Supplement: Supplementary Figure 4 — The influence of microglia elimination on the synapses in RCS rats or photoreceptors in control rats. (A,B) Confocal images of CtBP2- (green) and mGluR6-immunoreactive (red) puncta in the retinas of RCS rats and RCS rats treated with PLX3397 at P20 and P40. (C) Immunostaining for CtBP2 (green), PKCα (red), and mGluR6 (blue) in the retinas of control rats treated with PLX3397 at P40. (D) Representative high-resolution confocal images showing photoreceptor apoptosis using TUNEL (green) in the retinas of control rats and control rats treated with PLX3397 at P40. (E,F) An analysis CtBP2 and mGluR6 mRNA expression in the retinas of control rats, control rats treated with PLX, RCS rats, and RCS rats treated with PLX at P30 suggested that PLX had no effect on CtBP2 and mGluR6 expression (N = 3 rats per group). ONL, outer nuclear layer; OPL, outer plexiform layer; INL, inner nuclear layer; IPL, inner plexiform layer; GCL, ganglion cell layer. Scale bar, 50 μm (A-D). Bars represent means; error bars represent SD. using two-way ANOVA (E,F). [file Image_4.TIF]

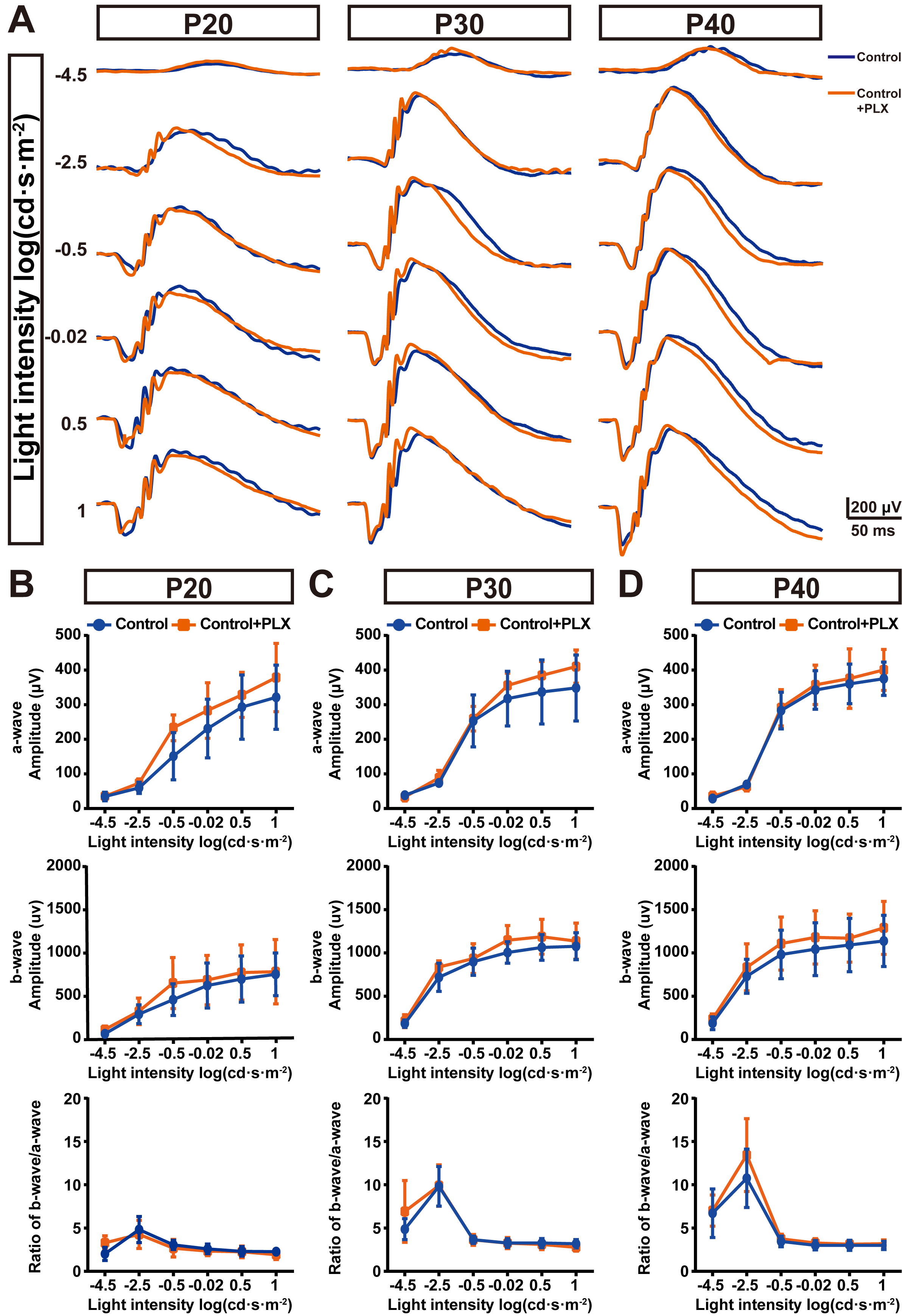

Supplement: Supplementary Figure 5 — Effects of microglia eliminate on the amplitudes of ERG signals in control rats. (A) Representative light-evoked ERG waveforms measured with six different light intensities [from -4.5 to 1 log(cd.s.m-2)] in P20, P30, and P40 control rats and control rats treated with PLX3397. (B-D) Average stimulus-response curves for a-wave amplitudes (top row), b-wave amplitudes (middle row) and the ratio of a-wave/b-wave amplitudes in control rats and control rats treated with PLX3397 at P20 (n = 14 and 8 rats, respectively), P30 (n = 20 and 10 rats, respectively), and P40 (n = 6 and 6 rats, respectively). Bars represent means; error bars represent SD. using two-way ANOVA (B-D). [file Image_5.TIF]
